# Evaluating the impact of park renovation on park-based physical activity: a natural experiment in Belgium with two years of follow-up

**DOI:** 10.1186/s12966-025-01846-0

**Published:** 2025-12-05

**Authors:** Amber Van Puyvelde, Jelle Van Cauwenberg, Jenny Veitch, Anna Timperio, Delfien Van Dyck, Louise Poppe, Benedicte Deforche

**Affiliations:** 1 Department of Public Health and Primary Care, Faculty of Medicine and Health Sciences, Ghent University, Ghent, Belgium; 2 Department of Movement and Sports Sciences (GE30), Faculty of Medicine and Health Sciences, Research Group Physical Activity and Health, Ghent University, Watersportlaan 2, Ghent, Belgium 9000; 3 Institute for Physical Activity and Nutrition (IPAN), School of Exercise and Nutrition Sciences, Deakin University, Geelong, Australia; 4 School of Public Health, Université Libre de Bruxelles, Brussels, Belgium; 5 Department of Movement and Sport Sciences, Vrije Universiteit Brussel, Brussel, Belgium

**Keywords:** Green space, Public open space, Natural environment, Long-term effect, Park improvement

## Abstract

**Background:**

Parks are valuable resources to promote physical activity (PA) among all ages. However, studies investigating the longer-term effect of park renewal on park-based PA are scarce and have not been conducted in Europe. This study builds upon prior research investigating the immediate effects of a total park renewal in Belgium on park-based PA. Specifically, it examines the overall and age-specific effects of the renewal one year and two years after its completion, providing insights into the longer-term impact on park-based PA.

**Methods:**

A natural experiment was conducted by observing the behaviour of the park visitors in two urban public parks (i.e., intervention and control park). The intervention involved extensive park renewal, including increased size, adding outdoor fitness equipment, a multi-use sports cage, six playgrounds targeting children of different ages, various benches, wheelchair-accessible picnic tables, wide concrete pathways, and removal of low vegetation. Observations were performed before, immediately after, and one and two years after the renewal using the System for Observing Play and Recreation in Communities. The number of visitors observed sedentary, walking or vigorously active were recorded according to age group and mean PA intensity levels were calculated. General(ized) linear models were fitted to assess the effect of the renewal on visitors’ PA in the park.

**Results:**

Overall, an increase was observed in the total number of visitors engaged in sedentary and vigorous activities one and two years after the renewal. However, no evidence for effects were found for the total number of visitors observed walking or for the mean PA intensity levels of the visitors. There were some age-specific differences: the number of adults and older adults observed sedentary increased, whereas no such effect was found for children or adolescents. Positive effects were observed for the number of adolescents, adults and older adults observed walking. Additionally, the number of children, adolescents and older adults engaging in vigorous PA increased in the intervention park compared to the control park.

**Conclusion:**

Extensive urban park renewal can increase both sedentary and active visitor numbers one and two years post-renewal, although effects vary across age groups.

**Supplementary Information:**

The online version contains supplementary material available at 10.1186/s12966-025-01846-0.

## Background

Worldwide, 81% of the adolescents and 31% of adults fail to meet the World Health Organization’s (WHO) guidelines on physical activity (PA) [1–3]. However, being physically active offers significant health benefits for individuals of all ages [4]. To tackle physical inactivity, a key action in the WHOs global plan is to enhance access to high-quality public and green open spaces [5]. Increasing access to green spaces, such as parks, could contribute to the promotion of PA as parks are typically free, accessible to all and provide pleasant environments for engaging in physical activities (e.g., biking, walking) [6]. However, evidence regarding the effect of park access on park-based PA levels is mixed [7–11], suggesting that other factors beyond park access, such as park amenities, may play an important role in the promotion of PA. Park renewals including improvements in park amenities and aesthetics such as upgrading paths and seating areas, and adding exercise equipment, have been shown to promote park-based PA [12]. However, more research is necessary to establish causal effects of changes to parks on PA levels [13–15]. Experimental research, especially randomized controlled trials (RCTs), are considered the gold standard to examine causal relations. However, when the intervention involves modifications to the built environment such as a park renewal, RCTs are not feasible as researchers are not able to randomize individuals’ exposure to the intervention. Hence, robust natural experimental designs provide a valuable alternative to assess the impact of changes to the built environment on PA.

Previous natural experiments examining the impact of park renewal on park-based PA have mainly been conducted in Australia and the USA, employing varied methodologies and including different types of refurbishments. Three natural experiments used a pre-post design without follow-up measurements to assess the longer term impact of the park improvements [16–18], with one of these lacking a control park [18]. Five natural experiments included a pre-test, post-test and one follow-up measurement [19–23], with all but one study [22] including a control park. Follow-up measurements were conducted 12 months [22, 23], 15 months [19] and 24 months [20, 21] after renovations were completed. The type of refurbishments varied across studies, ranging from the installation of new outdoor fitness equipment [19, 20, 22], seating [20], (nature-based) playgrounds [20, 21, 23], walking paths [20, 23] and picnic or BBQ areas [20, 23] to the addition of leash-free dog zones [23], landscaping [21, 23] and fencing [23].

These natural experiments yielded mixed results depending on age group and intervention scale. A North-American study found no significant PA changes following the installation of a new fitness area compared to a control park [19]. In contrast, an Australian study without a control park reported increased moderate-to-vigorous PA (MVPA) among children and male (older) adults three months after the installation of a new fitness area. However, at the 12 months follow-up, only the number of female older adults engaging in MVPA had significantly increased compared to baseline [22]. Three additional Australian studies found an increase in the total number of park visitors engaged in MVPA in the intervention parks compared to control parks [20, 21, 23]. The first study found an increase in the total number of visitors walking and the total number of visitors engaged in vigorous activities between pre-test and the 12 months follow-up [23]. No age-specific effects were investigated in this study [23]. Results of the second Australian study indicated an increase in the number of children and adults engaged in MVPA from pre-test to post-test (12 months later), while from pre-test to 24 months follow-up, only an increase in the number of children engaged in MVPA was observed [21]. In the third study [20] in the park with the most extensive renovations, the number of children and adults engaged in MVPA increased from pre-test to eight months post-test, and the number of children and older adults engaged in MVPA increased from pre-test to 24 months follow-up. In the park with less extensive refurbishment, the number of adolescents and adults engaged in MVPA decreased, as well as a decrease in the mean PA intensity levels of the park visitors [20]. Natural experiments investigating the effects of park renewal on park-based PA by age group are scarce and have exclusively been conducted in Australia [20–22]. Different ages have different needs and preferences for park features, and more research is necessary to investigate how a park renewal affects the PA levels in these different age groups. This could lead to a better understanding of which age groups benefit most from the renewal and addition of certain park features, and whether a park renewal equitably promotes PA across different demographic groups. Moreover, it strengthens the scientific evidence base by moving beyond aggregated effects and supports the development of more tailored and inclusive park design strategies that address the diverse needs of all age groups.

In summary, there is a lack of European studies examining the longer-term impact of park renewal on park-based PA, as previous natural experiments have been conducted exclusively in Australia and the USA. In Europe, urban parks are typically smaller in size but, they are more numerous and more widely distributed. In contrast, parks in the United States and Australia tend to be larger in scale but fewer in number [8]. More European natural experiments are needed to assess whether results from natural experiments in Australia and the USA can be generalised to Europe. Previous natural experiments included a maximum of two post-intervention measurements and most did not examine effects on PA by age group. To better understand the longer-term impact of costly and long-lasting park refurbishments on park users’ PA, more natural experiments with a robust study design are needed. These should include repeated measurements post refurbishment [24] and explore effects by age group. The current study addresses these gaps by examining the overall effect of a complete park renewal in Belgium on park-based PA one year and two years after the completion of the refurbishments. It builds upon prior research by Poppe et al. [25], which investigated the immediate effects of the park renewal by comparing park-based PA before and immediately after the renewal compared to a control park. That study found increases in the total number of sedentary, walking and vigorously active park visitors in the intervention park, but no evidence for changes in the mean PA levels of the park visitors, when compared to the control park [25]. The current study extends this research by incorporating two more follow-up measurements (i.e., one and two years post renewal) as well as new analyses investigating the effects of park renewal on park-based PA by age group.

## Methods

### Study setting

The study setting has previously been described by Poppe et al. [25] and by Van Puyvelde et al. [26]. This natural experiment was conducted in two urban parks in Ghent, Belgium. Ghent is a provincial capital with a population of approximately 270 000 inhabitants, a population density of 1699 inhabitants/km^2^ and a surface area of 156,2 km^2^. In Ghent, 19% of the population are children and adolescents (0–19 years), 64% are adults (20–59 years) and 17% are older adults (≥ 60 years) [27]. In 2014, the city council of Ghent selected the urban park ‘*De Vijvers*’ for renewal. The park is situated in a residential area and comprised a pond, walking trails, a grassy and a wooded area and a playground for children. Park ‘*Vaarnewijkpark*’ was selected as the control park due to its similarity in features and amenities to park ‘*De Vijvers’*, and absence of plans to renew the park during the study period. The parks are located on opposite sides of the city, 7,5 km walking distance from each other, which minimized the likelihood that changes to the intervention park would influence park use in the control park. Characteristics of the neighbourhoods in which the parks are located (e.g. residential density) are presented elsewhere [14, 15]. Furthermore, while both parks had similar amenities and characteristics at pre-test, the neighbourhoods in which they were located differed in their socio-economic characteristics (e.g., intervention park neighbourhood had lower income level and higher building density than control park neighbourhood). However, since both neighbourhoods showed similar socio-economic developments over time, these are unlikely to have substantially influenced changes in park visitation [16]. At pre-test, park ‘*De Vijvers’* was 51 313 m² whereas park ‘*Vaarnewijkpark’* was 31 502 m². This difference in size was accounted for in the analyses (see statistical analyses section).

The renewal of park ‘*De Vijvers’* was completed in January 2020. The renewal included repurposing a part of the parking lot from the adjacent elderly care facility which increased the park’s area by 12 000 m², resulting in a total park size of 63 313 m². This expansion enhanced park accessibility for residents of the care facility and their visitors. The renewal aimed to promote a range of uses of the park via social, active and relaxing park activities for various age groups and included removing low-growing vegetation, installing wide concrete pathways accessible for individuals with disabilities, adding picnic tables with wheelchair cutouts, and introducing new amenities such as outdoor fitness equipment, a multi-use sports cage, six playgrounds for different age groups (i.e., toddlers, children, and adolescents), and various benches. Detailed reports on the features and amenities of both parks before and after renewal have been published [25, 28] and images are available at the Open Science Framework (OSF) (https://osf.io/2z7sg/). To address the specific needs of older adults, the park renewal plans were co-designed with stakeholders from the adjacent elderly care facility (e.g., director and physiotherapist) [29]. At all times, both parks were accessible 24 hours/day and free of charge.

### Measures

The number and characteristics of the park visitors and their PA levels were observed using the System for Observing Play and Recreation in Communities (SOPARC). SOPARC is a reliable and feasible observation instrument for assessing PA and socio-demographic characteristics in community settings such as parks [30]. Visitor numbers and characteristics were recorded at both parks prior to the renewal of the intervention park (pre-test, July-October 2014), and at three post-renewal intervals: immediately following renewal (post-test, July-October 2020, six months post-intervention), one year later (follow-up one, July-October 2021, 18 months post-intervention), and two years later (follow-up two, July-October 2022, 30 months post-intervention). No changes were made to the control park between pre-test and follow-up two, or to the intervention park between post-test and follow-up two. As previously described [25, 26], the parks were divided into specific target areas, and observations were conducted on five non-rainy weekdays and four non-rainy weekend days, at four different times per day (i.e., observation moments; 7:30 AM, 12:30 PM, 3:30 PM, and 6:30 PM). Due to rain during some observation moments at pre- and post-test, additional observation days were scheduled (see Table 1), and the number of observation moments was factored into the analyses (see statistical analyses section). For each observation moment, researchers scanned all target areas and noted the number of visitors in each area based on estimated age group (children = 0–12 years, adolescents = 13–20 years, adults = 21–59 years, and older adults = ≥ 60 years) and activity level. Possible activity levels according to SOPARC were sedentary (including sitting, lying down and standing), walking or vigorous PA (e.g., running, playing, etc.). Data on age category and activity level of the park visitors were subsequently used to construct the dependent variables for statistical analyses (see Statistical Analyses section). Each observation moment took approximately 20–30 min. Observation moments were conducted simultaneously in both parks by a single researcher in each park. At post-test, a 3000 m² section of the renewed park was closed due to Covid-19 restrictions and thus not included in the observations, which was accounted for in the analyses (see statistical analyses section). This area was fully accessible and included in the observations during both follow-up periods. The observations were conducted by different researchers at all timepoints, with only one researcher remaining the same during post-test and follow-up one. However, all researchers received training with the SOPARC protocol and videos, and interrater reliability was assessed based on 20 test observation moments (10 in each park), with researchers independently recording visitor characteristics and PA levels at the same time and location. The intraclass correlation coefficient (ICC) was calculated to evaluate reliability. ICC values between 0.60 and 0.74 indicate good reliability, while values above 0.75 indicate excellent reliability [31]. Interrater reliability was found to be excellent across all timepoints (Table 2).

**Table 1:** Number of observations, and weather conditions

	Pre-test	Post-test	Follow-up 1	Follow-up 2
Weekdays with observations	6*	6**	5	5
Weekend days with observations	4	4	4	4
Observation moments across all days and both parks	72	80	72	72
Mean daily temperature (°C)	20	15	15	19

Each observation day consisted of four observation moments. One observation moment refers to a complete scan of all target areas in one park

*Due to rain, two observation moments were cancelled and performed on another weekday. The total number of observation moments was included in the analyses

** Due to rain, one extra non-rainy observation day (= four extra observation moments per park) was added. The total number of observation moments was included in the analyses

**Table 2:** Inter-rater reliability for number of observed visitors in each age and activity category

	Intraclass correlation coefficient (ICC)			
	Pre-test	Post-test	Follow-up 1	Follow-up 2
Children	0.97	1.00	0.86	0.99
Adolescents	0.99	0.92	0.91	0.97
Adults	0.98	0.96	0.93	0.96
Older adults	0.94	0.86	0.91	0.99
Visitors observed sedentary	0.96	0.97	0.93	0.99
Visitors observed walking	0.94	0.96	0.90	0.95
Visitors observed engaged in vigorous physical activity	0.99	0.96	0.90	0.96

### Statistical analyses

All data were analysed using R version 4.3.3 [32], following the same approach described by Poppe et al. [25] and Van Puyvelde et al. [26]. The data were aggregated for each observation moment per day for each timepoint (i.e., pre-test, post-test, follow-up one and two), and the total number of park visitors and the number of park visitors per activity level and age group were calculated. This resulted in 15 dependent variables for the analysis, namely the total number of visitors observed sedentary, walking and engaged in vigorous PA, respectively, and the number of children, adolescents, adults and older adults respectively observed engaging in each type of activity. To account for the skewed data distribution (i.e., excess amount of zero observations) and variations in the number of observation moments across the pre-test, post-test, and both follow-ups, the median number of visitors per observation moment were calculated for descriptive statistics. Differences in park size, including the difference between the two parks and the difference in size of the intervention park between the pre-test, post-test, and both follow-ups were taken into account by dividing the total number of visitors and visitors in each category (i.e., age, activity level) per observation moment by the park size (in ha) at the corresponding time [25]. To calculate the mean PA intensity of the park visitors, weighted metabolic equivalent (MET) scores were assigned to each activity level: 1 MET for sedentary, 3 METs for walking, and 6 METs for vigorous activity. These weighted MET scores were then multiplied by the observed number of visitors in each activity level category, summed, and, to obtain a mean PA intensity score independent of the number of visitors, this MET score was divided by the total number of park visitors observed during each time moment [25, 33]. This resulted in five more dependent variables for analysis, namely the mean PA intensity level of all park visitors (irrespective of age category) and the mean PA intensity levels for children, adolescents, adults and older adults.

To address the issue of multiple testing, omnibus likelihood ratio tests (i.e. type II ANOVA with main effects of park and time, and their interaction) were performed. Doing so allowed us to focus on the park-by-time interactions that were identified by the omnibus tests as relevant and hence reduce the number of reported tests. The results of the omnibus likelihood ratio tests are presented in Supplementary file 1. Where a statistically significant park-by-time interaction was observed (i.e., p-value for the omnibus test ≤ 0.05), post hoc analyses were performed to examine intervention effects between timepoints (i.e., between pre-test and post-test; pre-test and follow-up 1; pre-test and follow-up 2; post-test and follow-up 1; post-test and follow up 2; and follow-up 1 and follow-up 2), with results presented in the manuscript. Post hoc analyses for non-significant omnibus tests are included in Supplementary files 2–3, but should be interpreted with caution due to the higher likelihood of Type II errors.

Eleven of the 20 dependent variables had ≥ 10% zero values (see Table 3), indicating that no visitors in the specified category were observed during several observation moments. These zero-inflated variables were analysed using hurdle models, while non zero-inflated variables were analysed using general(ized) linear models. A hurdle model comprises two components. In the first part, the likelihood of not obtaining a zero value, representing the probability of observing any visitors from the specified category during an observation moment, was estimated using logistic regression. In the second part, a regression was performed on non-zero observations, providing the average difference between the intervention and control park in visitor changes between two timepoints when at least one visitor of the specified category was observed at one of the two timepoints. This resulted in 31 models that were run to analyse the 20 dependent variables (see Supplementary file 1; 11 hurdle models and nine general(ized) linear models). Combined with the omnibus likelihood ratio tests described above, the reported tests were reduced from 186 (31 models multiplied with 6 post-hoc analyses) to 66 (11 models with significant results for the omnibus likelihood ratio test multiplied with 6 post-hoc analyses). Due to the clustering of observation moments within days, analysis of variance was used to compare an empty model with a fixed intercept against one allowing varying intercepts for observation day. For all variables, the fixed-intercept model performed better, thus general(ized) linear fixed intercept models were used for all variables. Models with different variance and link functions (Gaussian with identity, Gamma with log, and Gamma with identity) were compared using Akaike Information Criterion (AIC), and the model with the lowest AIC was selected. For interpretability, beta coefficients of models with a Gamma distribution and log link were exponentiated. As quasi-complete separation was detected in the logistic part of the hurdle model of the variable ‘adult visitors observed sedentary’, Firth’s modified score procedure was applied to correct the infinite parameter estimates. Homoscedasticity was assessed visually by plotting deviance residuals against the fitted values. Following recent recommendations [34], effect relevance was determined based on confidence interval (CI) estimates rather than p-value dichotomization.

## Results

### Descriptive statistics of PA levels of park visitors

The median and interquartile range (IQR) of the total number of observed park visitors in each activity level across all observation moments at pre-test, post-test, follow-up one and follow-up two are presented in Table 3. Due to the skewed data distribution and the difference in the number of observation moments per timepoint, the mean and standard deviation (SD) of the total number of observed park visitors in each activity level are presented in Supplementary file 4.

**Table 3:** Percentage of zero values for each dependent variable and median and IQR of the number of visitors per hectare according to activity level and timepoint

			Intervention Park				Control Park			
		% zero values	Pre-test Median (IQR)	Post-test Median (IQR)	Follow-up 1 Median (IQR)	Follow-up 2 Median (IQR)	Pre-test Median (IQR)	Post-test Median (IQR)	Follow-up 1 Median (IQR)	Follow-up 2 Median (IQR)
Total park visitors/hectare		0	1.85 (1.75)	4.56 (5.02)	5.61 (6.95)	4.90 (5.01)	2.38 (3.81)	1.59 (2.54)	1.59 (2.94)	2.22 (1.59)
Park visitors observed sedentary (n/hectare)										
Total		0	0.97 (0.97)	1.49 (2.24)	3.32 (3.95)	2.61 (2.49)	0.95 (1.27)	0.63 (0.63)	0.63 (0.63)	0.63 (0.95)
Children		49	0.00 (0.19)	0.33 (0.99)	0.47 (0.63)	0.24 (0.63)	0.00 (0.00)	0.00 (0.32)	0.00 (0.00)	0.00 (0.32)
Adolescents		63	0.00 (0.39)	0.00 (0.33)	0.16 (0.47)	0.00 (0.12)	0.00 (0.32)	0.00 (0.63)	0.00 (0.00)	0.00 (0.00)
Adults		16	0.39 (0.49)	0.66 (0.91)	1.90 (1.58)	1.58 (1.38)	0.32 (0.95)	0.32 (0.48)	0.32 (0.48)	0.63 (0.32)
Older adults		61	0.00 (0.05)	0.17 (0.17)	0.16 (1.11)	0.32 (1.11)	0.00 (0.00)	0.00 (0.00)	0.00 (0.00)	0.00 (0.00)
Park visitors observed walking (n/hectare)										
Total		0	0.58 (0.58)	1.16 (1.24)	1.58 (1.58)	0.95 (0.63)	0.63 (0.63)	0.63 (0.95)	0.63 (0.63)	0.63 (0.95)
Children		68	0.00 (0.00)	0.17 (0.33)	0.00 (0.47)	0.00 (0.04)	0.00 (0.00)	0.00 (0.32)	0.00 (0.00)	0.00 (0.00)
Adolescents		68	0.00 (0.19)	0.00 (0.17)	0.16 (0.32)	0.00 (0.00)	0.32 (0.32)	0.00 (0.32)	0.00 (0.32)	0.00 (0.00)
Adults		14	04.39 (0.39)	0.83 (0.83)	0.95 (1.26)	0.79 (0.47)	0.32 (0.63)	0.32 (0.40)	0.32 (0.32)	0.63 (0.63)
Older adults		57	0.00 (0.19)	0.17 (0.17)	0.16 (0.24)	0.00 (0.20)	0.00 (0.32)	0.00 (0.00)	0.00 (0.00)	0.00 (0.32)
Park visitors observed engaged in vigorous PA (n/hectare)										
Total	0		0.78 (0.78)	2.07 (1.70)	1.42 (1.74)	1.60 (1.34)	1.43 (2.46)	1.27 (1.59)	0.95 (1.75)	0.95 (0.79)
Children	54		0.00 (0.19)	0.25 (0.66)	0.32 (1.03)	0.32 (0.63)	0.00 (0.71)	0.00 (0.32)	0.00 (0.32)	0.00 (0.32)
Adolescents	54		0.00 (0.19)	0.17 (0.50)	0.17 (0.47)	0.00 (0.24)	0.00 (0.71)	0.00 (0.32)	0.00 (0.40)	0.00 (0.32)
Adults	8		0.58 (0.58)	0.99 (0.66)	0.79 (0.47)	0.95 (1.11)	0.32 (0.71)	0.63 (0.95)	0.63 (0.95)	0.63 (0.63)
Older adults	53		0.00 (0.19)	0.17 (0.33)	0.00 (0.16)	0.16 (0.32)	0.16 (0.32)	0.00 (0.32)	0.00 (0.32)	0.00 (0.32)

*IQR* = Interquartile range, *PA =* physical activity

The mean and SD for the weighted MET scores of the park visitors for the total sample and the four age groups separately are reported in Table 4. The mean scores ranged between 2.61 (SD = 0.93) for older adults in the intervention park at follow-up one to 5.21 (SD = 1.63) for adolescents in control park at follow-up two. This indicates that on average, park visitors of all ages in both parks were engaging in light to moderate PA.

**Table 4:** Mean and SD for the weighted MET scores of the park visitors

	Intervention Park				Control Park			
	Pre-test Mean (SD)	Post-test Mean (SD)	Follow-up 1 Mean (SD)	Follow-up 2 Mean (SD)	Pre-test Mean (SD)	Post-test Mean (SD)	Follow-up 1 Mean (SD)	Follow-up 2 Mean (SD)
Total	3.95 (1.19)	3.86 (0.80)	3.29 (0.68)	3.46 (1.05)	4.00 (1.37)	4.17 (1.22)	4.20 (1.22)	4.05 (0.96)
Children	3.70 (1.87)	3.38 (1.20)	3.64 (1.50)	3.64 (1.26)	4.77 (1.53)	3.32 (1.76)	4.01 (1.85)	3.43 (1.93)
Adolescents	3.85 (1.77)	3.99 (1.75)	3.64 (1.31)	4.17 (1.84)	3.75 (1.76)	3.58 (1.70)	4.63 (1.72)	5.21 (1.63)
Adults	4.04 (1.35)	3.87 (0.82)	3.22 (0.92)	3.41 (1.24)	4.05 (1.84)	4.32 (1.45)	3.99 (1.46)	3.94 (1.19)
Older adults	3.75 (1.84)	3.85 (1.40)	2.61 (0.93)	3.21 (1.73)	4.18 (1.81)	4.58 (1.92)	4.69 (1.65)	4.01 (1.73)

*SD* = standard deviation, *MET score* = metabolic equivalent score

### Effect of park renewal on the number of park visitors observed sedentary

**Fig. 1:**
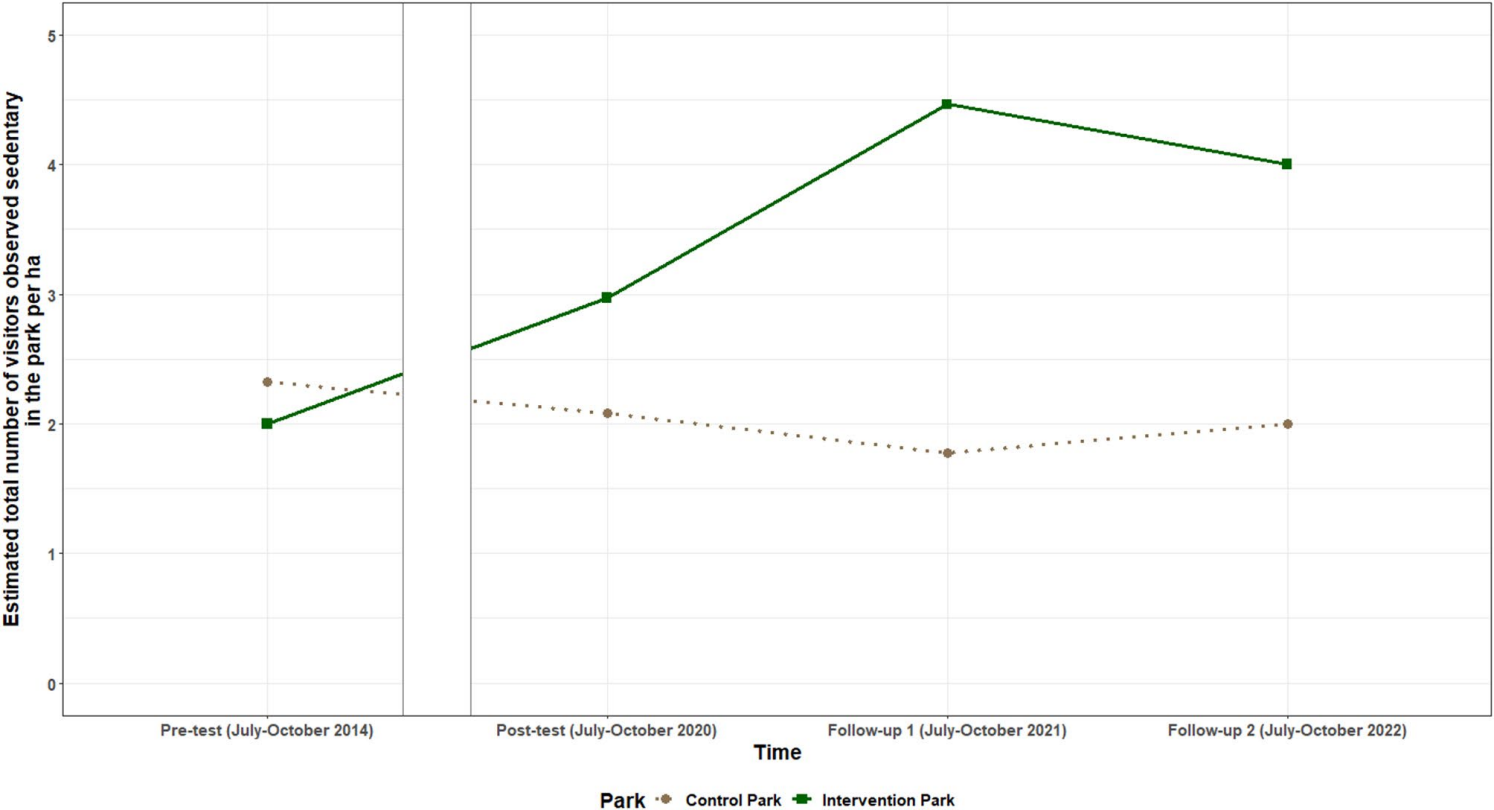
Estimated total number of visitors/ha observed sedentary per timepoint for the intervention and control park

Intervention effects were found for the **total number of park visitors observed sedentary** between pre-test and all post-intervention timepoints (i.e., post-test, follow-up one and two). The estimates of the total number of park visitors observed sedentary can be interpreted as the average difference between the intervention and control park in the change in number of visitors observed sedentary from pre-test to all post-intervention timepoints. Hence, the estimates reflect how the change in the number of park visitors observed sedentary/hectare over time differs between the intervention and the control park. For example, the total number of visitors observed sedentary increased from pre-test to post-test with on average 1.21 (95% CI: 0.25; 2.17) visitors/ha more in the intervention park. Between pre-test and follow-up one, this was on average 3.01 (95% CI: 1.83; 4.20) visitors/ha, and between pre-test and follow-up two this was 2.33 (95% CI: 1.19; 3.46) visitors/ha. The total number of park visitors observed sedentary increased between pre-test and all post-intervention timepoints, and also increased between post-test and follow-up one. The estimated total number of visitors/ha observed sedentary per timepoint for both parks is shown in Fig. 1.

**Fig. 2:**
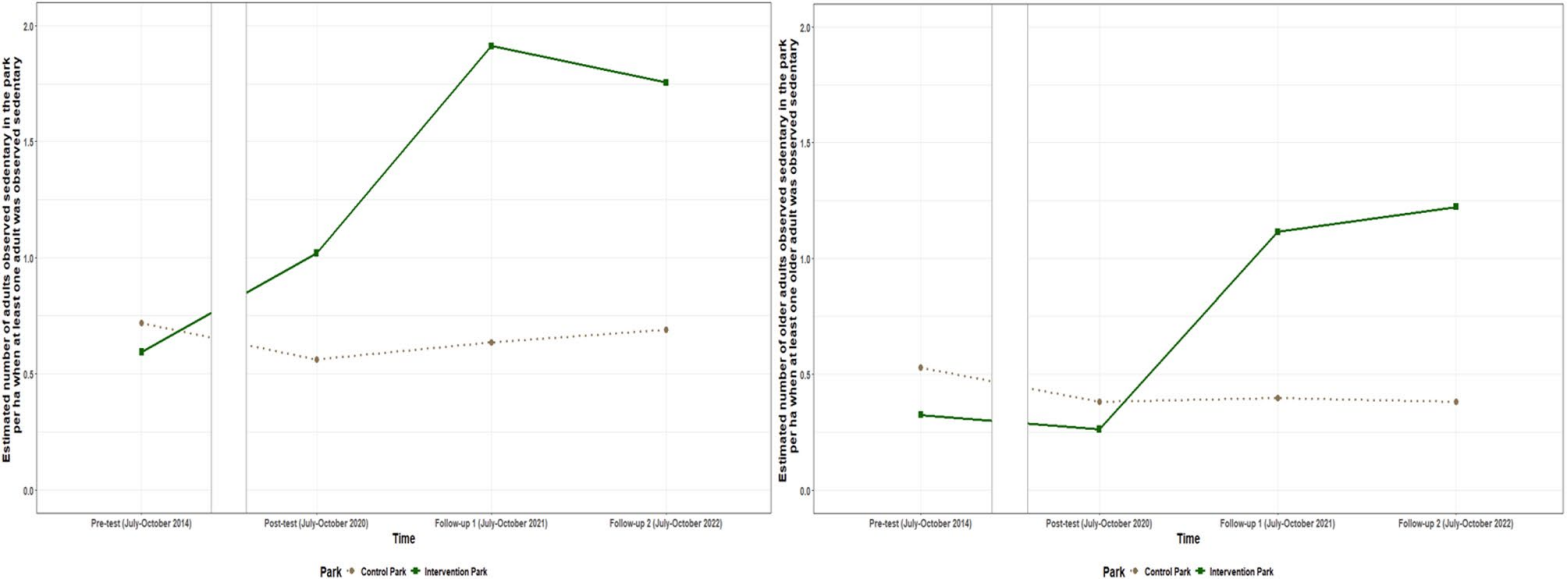
Estimated number of adult and older adult visitors/ha observed sedentary per timepoint for the intervention and control park when at least one adult or older adult was observed sedentary

The results for the number of adults and older adults observed sedentary are presented in Table 5. Among **adult visitors**, intervention effects for observed sedentary activities were found between pre-test and all post intervention timepoints. When at least one adult was observed sedentary in the park, the number of adults/ha observed sedentary increased in the intervention park compared to the control park by an average of 0.59 (95% CI: 0.08; 1.09) adults/ha from pre-test to post-test, 1.40 (95%CI: 0.68; 2.12) adults/ha from pre-test to follow-up one, and 1.19 (95% CI: 0.50; 1.88) adults/ha from pre-test to follow-up two. The number of adults/ha observed sedentary also increased between post-test and follow-up one. For **older adult visitors observed sedentary**, intervention effects were found between pre-test and both follow-ups, and between post-test and both follow-ups. The number of older adults observed sedentary increased in the intervention park, compared to the control park by an average of 0.92 (95% CI: 0.24;1.60) older adults/ha between pre-test and follow-up one, and 1.05 (95% CI: 0.36; 1.73) older adults/ha from pre-test to follow-up two. No intervention effects were found for the number of children or adolescents observed sedentary. The estimated number of adult and older adult visitors/ha observed sedentary per timepoint for both parks is shown in Fig. 2.

**Table 5:** Intervention effects for the number of visitors observed sedentary per hectare

	Intervention effects when at least one visitor of the specified category is observed		
	**Pre-test** – Post-test Time x Park beta (95% CI) *p* value	**Pre-test** – Follow-up 1 Time x Park beta (95% CI) *p* value	**Pre-test**– Follow-up 2 Time x Park beta (95% CI) *p* value
Adults^b^	**0.59 (0.08; 1.09)*p* = 0.02**	**1.40 (0.68; 2.12) *p* < 0.001**	**1.19 (0.50; 1.88) *p* < 0.001**
Older adults^b^	0.09 (−0.43; 0.60) *p* = 0.74	**0.92 (0.24; 1.60) *p* = 0.01**	**1.05 (0.36; 1.73) *p* = 0.003**

	**Post-test** **– Follow-up 1** Time x Park beta (95% CI) *p* value	**Post-test** **– Follow-up 2** Time x Park beta (95% CI) *p* value	**Follow-up 1** **– Follow-up 2** Time x Park beta (95% CI) *p* value
Adults^b^	**0.82 (0.10; 1.54) *p* = 0.03**	0.60 (−0.09; 1.29) *p* = 0.09	−0.21 (−1.07; 0.64) *p* = 0.62
Older adults^b^	**0.84 (0.22; 1.45) ***p*** = 0.008**	**0.96 (0.34; 1.58) ***p*** = 0.003**	0.12 (−0.64; 0.89) *p* = 0.75

Time by park interaction with ‘park = control’ and underlined time moment as reference categories. CI = confidence interval

^a^Regular Gamma model with identity link, ^b^Hurdle model with part one = logistic regression (not shown because omnibus likelihood ratio tests > 0.05) and part two = Gamma model with identity link. Bold results represent intervention effects

### Effect of park renewal on the number of park visitors observed walking

**Fig. 3:**
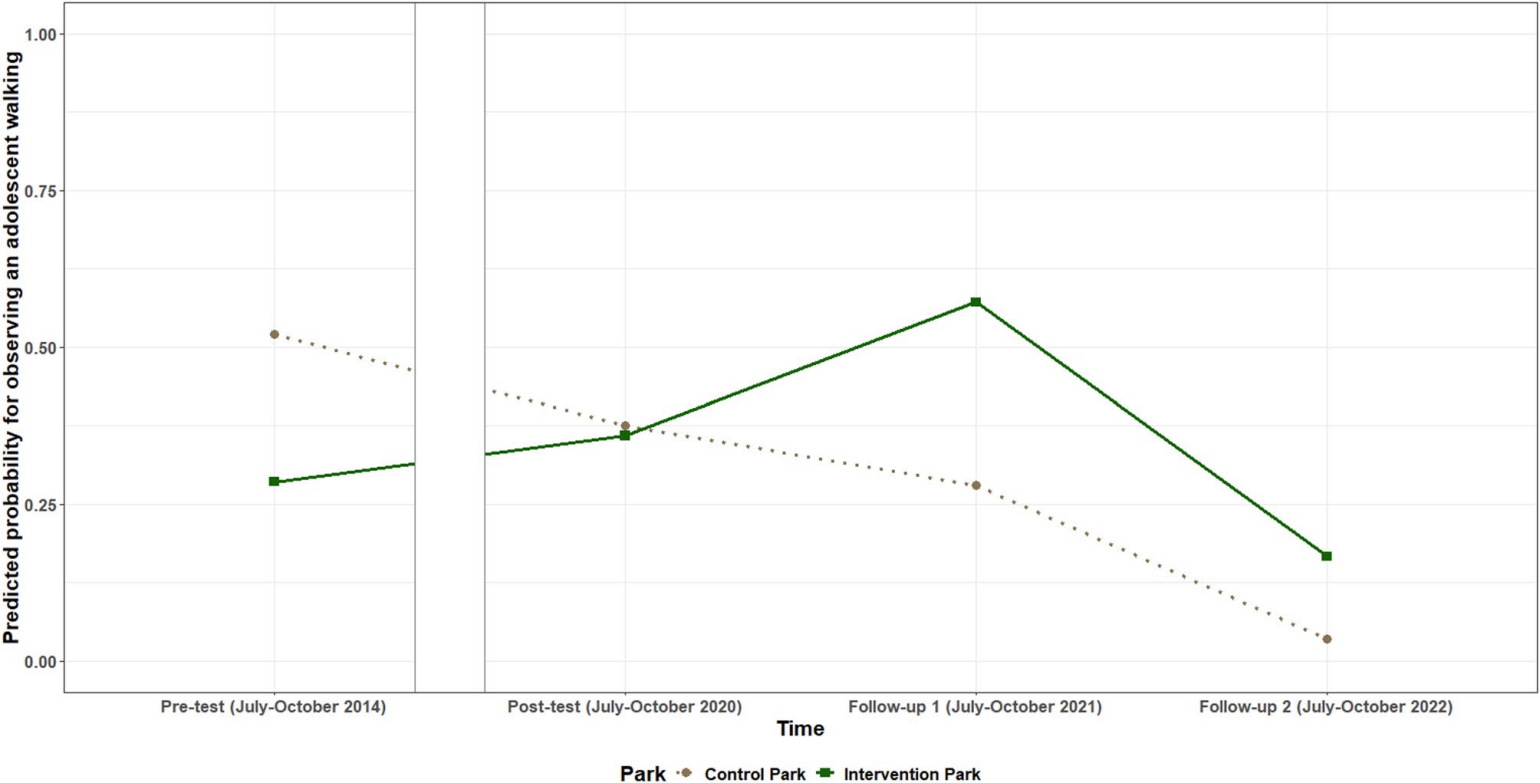
Estimated probability for observing an adolescent walking per timepoint in the intervention and control park

**Fig. 4:**
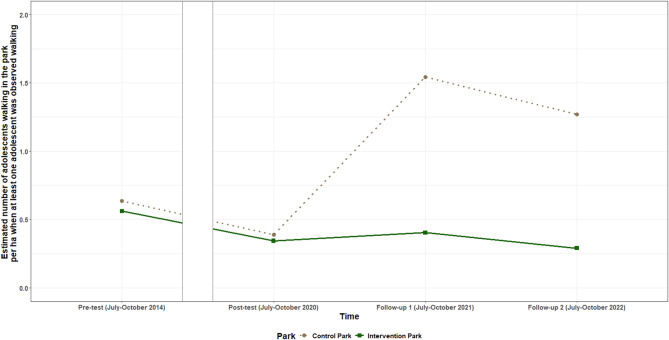
Estimated number of adolescent visitors/ha walking per timepoint for the intervention and control park when at least one adolescent was observed walking

Table 6 provides the intervention effects for the number of adolescents, adults and older adults observed walking. The logistic model provides the intervention effect for the odds of observing any walking visitor of the specified age category, while the gamma identity model provides the intervention effect when at least one visitor of the specified category was observed walking. **For adolescents**, the odds of observing anyone walking in the intervention park compared to the control park, increased from pre-test to both follow-up measurements. Between pre-test and follow-up one, the odds of observing at least one adolescent walking were on average 9.29 (95%CI: 1.91; 45.11) times higher for the intervention park compared to the control park. Between pre-test and follow-up two, the odds of observing at least one adolescent walking were on average 15.17 (95%CI: 1.30; 176.73) times higher for the intervention park compared to the control park. However, when at least one adolescent was observed walking in the park, the number of adolescents observed walking decreased by on average 1.06 (95% CI: −2.09; −0.04) adolescents/ha between pre-test and follow-up one in the intervention park, compared to the control park. The number of adolescents/ha observed walking also decreased in the intervention park between post-test and follow-up one, when compared to the control park. The estimated probability for observing an adolescent walking is shown in Fig. 3, while the estimated number of adolescent visitors/ha observed walking per timepoint for both parks is shown in Fig. 4.

**Table 6:** Intervention effects for the number of visitors observed walking

	Intervention effect for the odds of observing any walking visitor of the specified category			Intervention effect when at least one visitor of the specified category was observed walking		
	**Pre-test** – Post-test Time x Park OR (95% CI) *p* value	**Pre-test** – Follow-up 1 Time x Park OR (95% CI) *p* value	**Pre-test** – Follow-up 2 Time x Park OR (95% CI) *p* value	**Pre-test** – Post-test Time x Park beta (95% CI) *p* value	**Pre-test** – Follow-up 1 Time x Park beta (95% CI) *p* value	**Pre-test** – Follow-up 2 Time x Park beta (95% CI) *p* value
Adolescents^b^	2.53 (0.54; 11.90) *p* = 0.24	**9.29 (1.91; 45.11) *p* = 0.01**	**15.17 (1.30; 176.73) ***p*** = 0.03**	0.03 (−0.46; 0.52) *p* = 0.90	**−1.06 (−2.09; −0.04) *p* = 0.04**	−0.91 (−2.97; 1.16) *p* = 0.38
Adults^b^	1.93 (0.14; 26.52) *p* = 0.62	0.34 (0.03; 3.62) *p* = 0.37	0.68 (0.07; 6.19) *p* = 0.73	**0.43 (0.09; 0.78) *p* = 0.01**	**0.59 (0.21; 0.98) *p* = 0.003**	0.02 (−0.36; 0.40) *p* = 0.92
Older adults^b^	**6.67 (1.30; 34.24) *p* = 0.02**	**17.81 (3.36; 94.30) *p* < 0.001**	2.44 (0.53; 11.13) *p* = 0.25	0.37 (−0.09; 0.83) *p* = 0.11	0.22 (−0.25; 0.68) *p* = 0.36	0.31 (−0.09; 0.72) *p* = 0.13

	**Post-test** – Follow-up 1 Time x Park OR (95% CI) *p* value	**Post-test** – Follow-up 2 Time x Park OR (95% CI) *p* value	**Follow-up 1** – Follow-up 2 Time x Park OR (95% CI) *p* value	**Post-test** – Follow-up 1 Time x Park beta (95% CI) *p* value	**Post-test** – Follow-up 2 Time x Park beta (95% CI) *p* value	**Follow-up 1** – Follow-up 2 Time x Park beta (95% CI) *p* value
Adolescents^b^	3.67 (0.80; 16.86) *p* = 0.09	6.00 (0.53; 67.42) *p* = 0.15	1.63 (0.14; 18.73) *p* = 0.69	**−1.09 (−2.06; −0.13) *p* = 0.03**	−0.94 (−2.97; 1.10) *p* = 0.36	0.16 (−2.06; 2.38) *p* = 0.89
Adults^b^	0.18 (0.01; 3.16) *p* = 0.24	0.35 (0.02; 5.55) *p* = 0.46	1.97 (0.16; 24.4) *p* = 0.60	0.16 (−0.26; 0.59) *p* = 0.46	−0.41 (−0.83; 0.008) *p* = 0.05	**−0.57 (−1.03; −0.12) *p* = 0.01**
Older adults^b^	2.67 (0.50; 14.37) *p* = 0.25	0.37 (0.08; 1.70) *p* = 0.20	**0.14 (0.03; 0.66) *p* = 0.01**	−0.15 (−0.64; 0.33) *p* = 0.53	−0.06 (−0.48; 0.36) *p* = 0.79	0.09 (−0.34; 0.53) *p* = 0.66

Time by park interaction with ‘park = control’ and underlined time moment as reference categories

*OR =* odds ratio, *CI* =confidence interval

^b^Hurdle model with part one = logistic regression and part two = gamma model with identity link. Bold results represent intervention effects

**Fig. 5:**
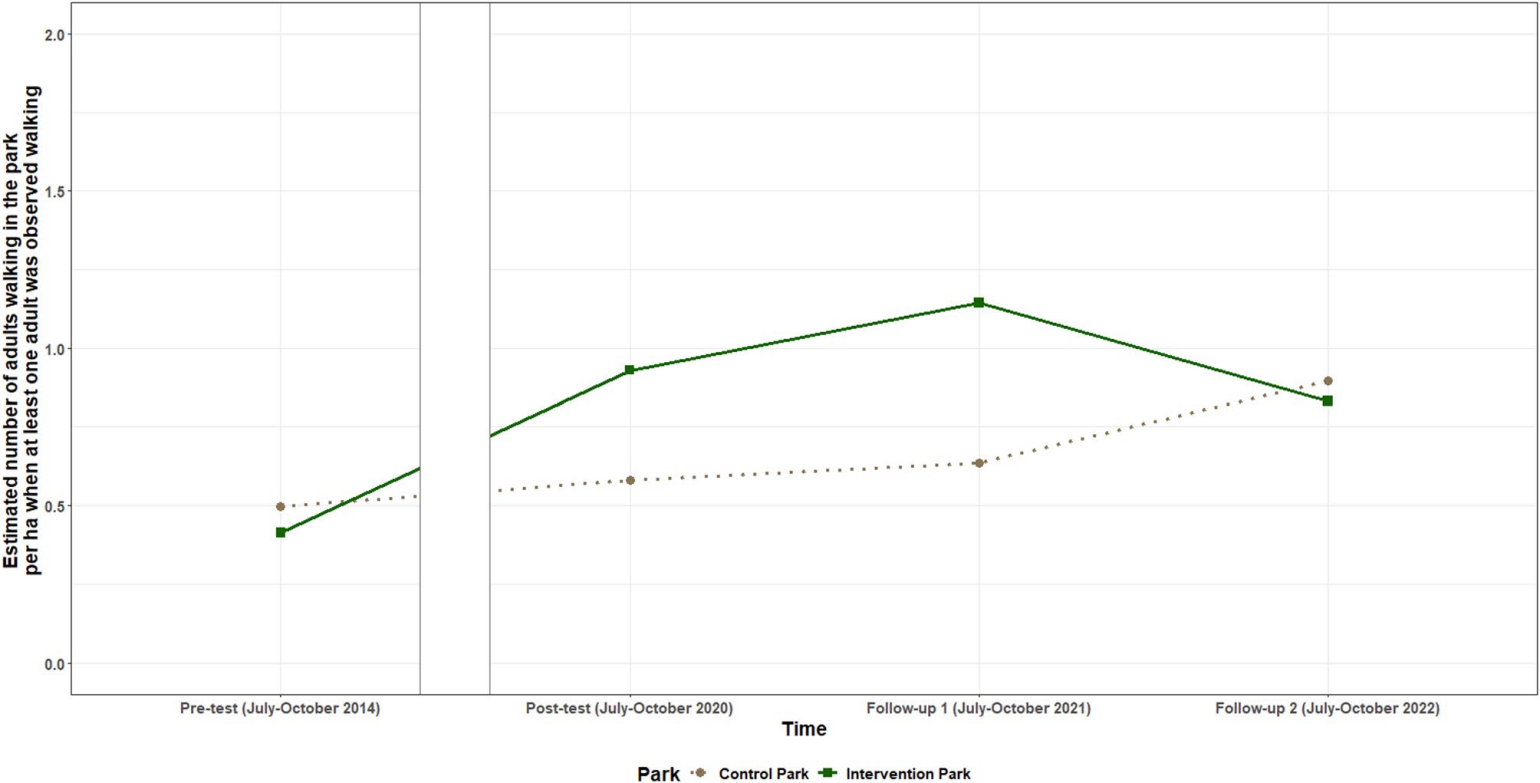
Estimated number of adult visitors/ha walking per timepoint for the intervention and control park when at least one adult was observed walking

**Fig. 6:**
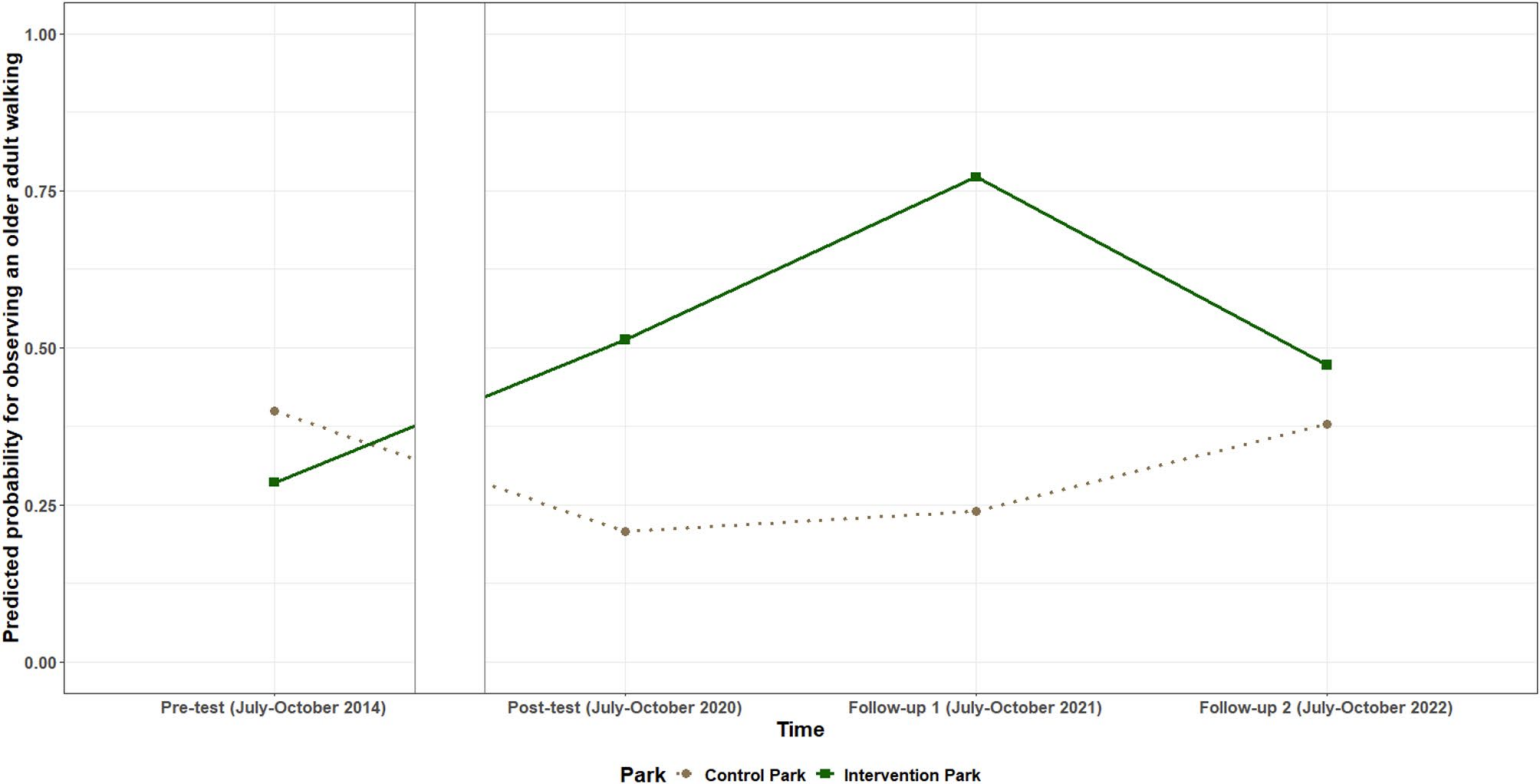
Estimated probability for observing an older adult walking per timepoint in the intervention and control park

Positive intervention effects were found for the number of adults and older adults observed walking between pre-test and post-test and pre-test and follow-up one. When at least one adult was observed walking in the park, **the number of adults observed walking** between pre-test and post-test increased by on average 0.43 (95% CI: 0.09; 0.78) adults/ha in the intervention park, compared to the control park, and by 0.59 (95% CI: 0.21; 0.98) adults/ha between pre-test and follow-up one. The odds of observing at least one **older adult walking** were on average 6.67 (95%CI: 1.30; 34.24) times higher between pre-test and post-test, and 17.81 (95%CI: 3.36; 94.30) times higher between pre-test and follow-up one for the intervention park, compared to the control park. In addition, a decrease was observed in the number of adult visitors/ha observed walking and the odds to observe any older adult walking in the intervention park between follow-up one and two, compared to the control park. The estimated number of adult visitors/ha observed walking per timepoint for both parks is shown in Fig. 5, while the estimated probability for observing an older adult walking is shown in Fig. 6. No intervention effects were found for the total number of visitors walking or the number of children walking.

### Effect of park renewal on the number of park visitors observed being vigorously active

**Fig. 7:**
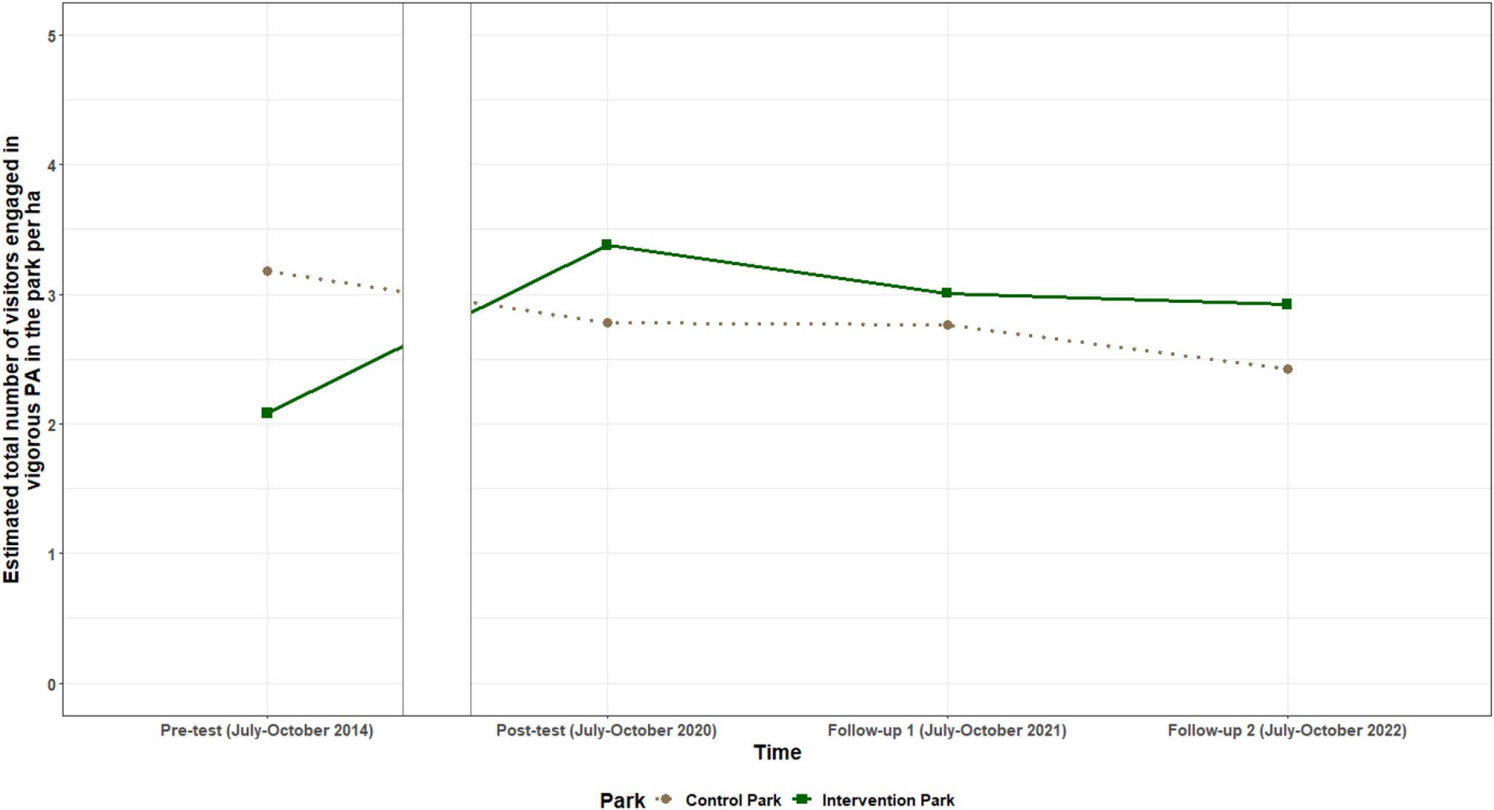
Estimated total number of visitors/ha engaged in vigorous PA per timepoint for the intervention and control park

Positive intervention effects were found for the **total number of visitors engaged in vigorous PA** between pre-test and post-test as well as both follow-ups. The total number of visitors engaged in vigorous PA increased from pre-test to post-test with on average 1.70 (95% CI: 0.62; 2.78) visitors/ha more in the intervention park, compared to the control park. Between pre-test and follow-up one, this was on average 1.34 (95% CI: 0.29; 2.40) visitors/ha, and between pre-test and follow-up two this was 1.60 (95% CI: 0.58; 2.62) visitors/ha. No intervention effects were found between any of the post-intervention timepoints. The estimated total number of park visitors/ha engaged in vigorous PA per timepoint for the intervention and control park are shown in Fig. 7.

**Fig. 8:**
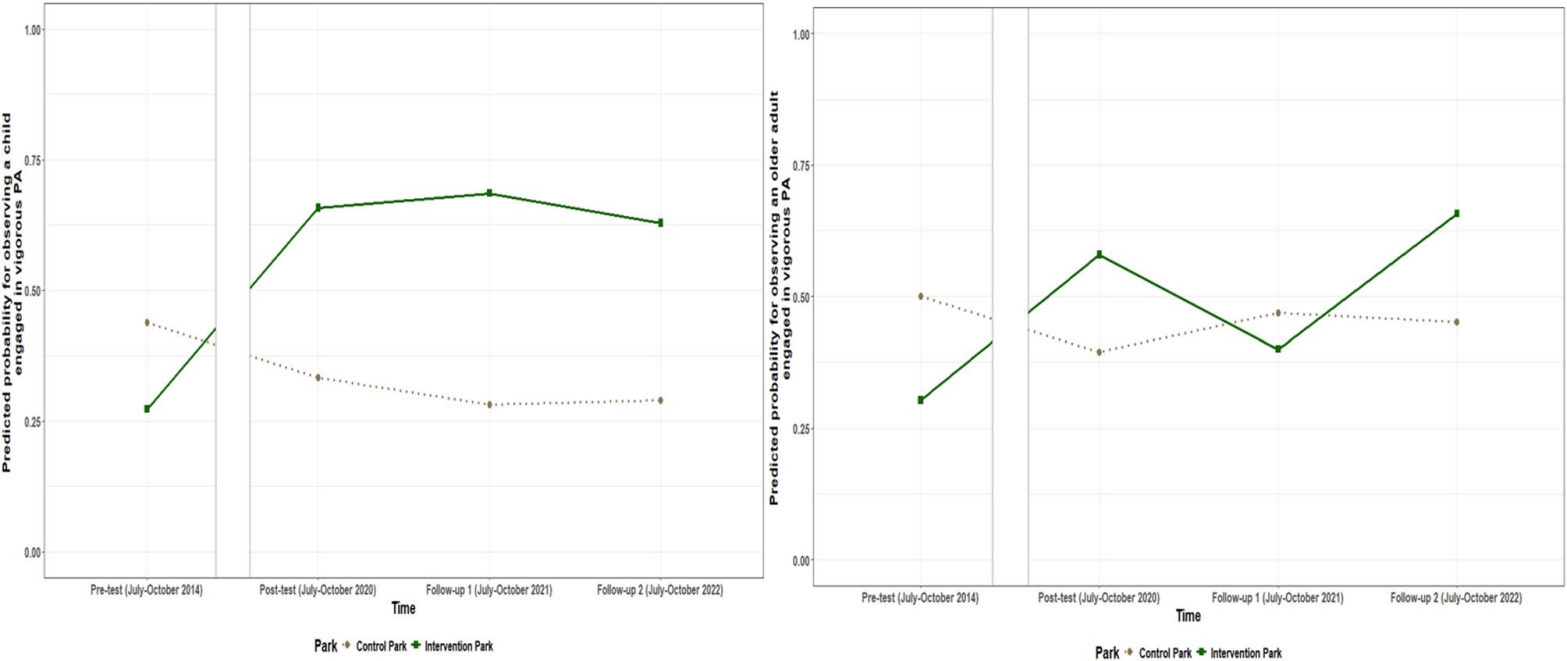
Estimated probability for observing a child and older adult engaged in vigorous PA per timepoint in the intervention and control park

**Fig. 9:**
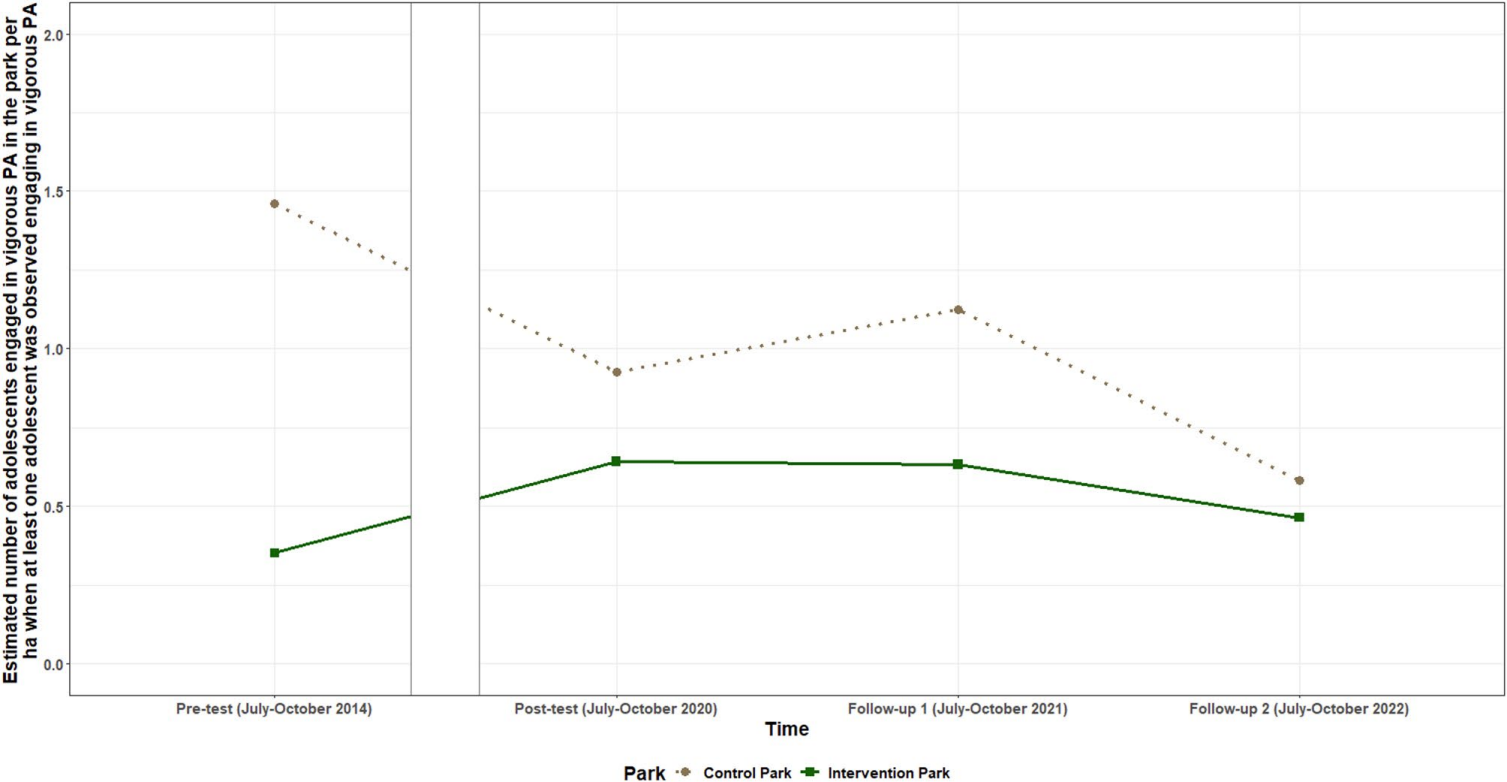
Estimated number of adolescent visitors/ha engaged in vigorous PA per timepoint for the intervention and control park when at least one adolescent was observed engaging in vigorous PA

The intervention effects for the number of children, adolescents, and older adults engaged in vigorous PA are presented in Table 7. The odds of observing any **child engaged in** **vigorous PA** increased in the intervention park, in comparison to the control park between pre-test and post-test and between pre-test and both follow-ups. The odds of observing at least one child engaged in vigorous PA were on average 7.97 (95%CI: 1.91; 33.37) times higher between pre-test and post-test, 11.56 (95% CI: 2.65; 50.59) times higher between pre-test and follow up one and 8.58 (95% CI: 1.98; 37.13) times higher between pre-test and follow-up two for the intervention park, compared to the control park. **The number of adolescents engaged in vigorous PA** increased between pre-test and follow-up two by on average 0.99 (95% CI: 0.24; 1.75) adolescents/ha in the intervention park, compared to the control park. The odds of observing any **older adults engaged in vigorous PA** increased in the intervention park in comparison to the control park between pre-test and post-test with on average 4.87 (95% CI: 1.21; 19.54) times and between pre-test and follow-up two with on average 5.35 (95% CI: 1.29; 22.16) times. No intervention effects were found between any of the post-intervention timepoints for these three variables or for the number of adults engaged in vigorous PA. The estimated probability for observing a child or older adult engaged in vigorous PA is shown in Fig. 8, while the estimated number of adolescent visitors/ha observed engaged in vigorous PA per timepoint for both parks is shown in Fig. 9.

**Table 7:** Intervention effects for the number of visitors observed engaging in vigorous PA

	Intervention effect for the odds of observing any visitor engaged in vigorous PA of the specified category			Intervention effect when at least one visitor of the specified category was observed engaged in vigorous PA		
	**Pre-test** – Post-test Time x Park OR (95% CI) *p* value	**Pre-test** – Follow-up 1 Time x Park OR (95% CI) *p* value	**Pre-test** – Follow-up 2 Time x Park OR (95% CI) *p* value	**Pre-test** – Post-test Time x Park beta (95% CI) *p* value	**Pre-test** – Follow-up 1 Time x Park beta (95% CI) *p* value	**Pre-test** – Follow-up 2 Time x Park beta (95% CI) *p* value
Children^b^	**7.97 (1.91; 33.37) ** ***p*** ** = 0.004**	**11.56 (2.65; 50.59) ** ***p*** ** = 0.001**	**8.58 (1.98; 37.13) ** ***p*** ** = 0.004**	0.35 (−0.99; 1.70) *p* = 0.60	0.68 (−0.63; 1.99) *p* = 0.31	0.80 (−0.27; 1.87) *p* = 0.14
Adolescents^b^	2.84 (0.72; 11.18) *p* = 0.14	1.64 (0.41; 6.48) *p* = 0.48	1.41 (0.35; 5.64) *p* = 0.63	0.83 (−0.02; 1.67) *p* = 0.06	0.62 (−0.28; 1.51) *p* = 0.17	**0.99 (0.24; 1.75)** ***p*** **= 0.01**
Older adults^b^	**4.87 (1.21; 19.54) ** *p* ** = 0.03**	1.74 (0.43; 7.07) *p* = 0.44	**5.35 (1.29; 22.16) ** *p* ** = 0.02**	0.04 (−0.16; 0.24) *p* = 0.72	−0.04 (−0.24; 0.17) *p* = 0.72	0.03 (−0.16; 0.22) *p* = 0.76

	**Post-test** – Follow-up 1 Time x Park OR (95% CI) *p* value	**Post-test** – Follow-up 2 Time x Park OR (95% CI) *p* value	**Follow-up 1** – Follow-up 2 Time x Park OR (95% CI) *p* value	**Post-test** – Follow-up 1 Time x Park beta (95% CI) *p* value	**Post-test** – Follow-up 2 Time x Park beta (95% CI) *p* value	**Follow-up 1** – Follow-up 2 Time x Park beta (95% CI) *p* value
Children^b^	1.45 (0.34; 6.12) *p* = 0.61	1.08 (0.26; 4.49) *p* = 0.92	0.74 (0.17; 3.24) *p* = 0.69	0.32 (−1.10; 1.74) *p* = 0.65	0.44 (−0.76; 1.65) *p* = 0.47	0.12 (−1.04; 1.28) *p* = 0.84
Adolescents^b^	0.58 (0.15; 2.26) *p* = 0.43	0.50 (0.13; 1.97) *p* = 0.32	0.86 (0.22; 3.42) *p* = 0.83	−0.21 (−0.99; 0.58) *p* = 0.60	0.16 (−0.46; 0.79) *p* = 0.60	0.37 (−0.32; 1.06) *p* = 0.29
Older adults^b^	0.36 (0.09; 1.39) *p* = 0.14	1.10 (0.28; 4.35) *p* = 0.89	3.08 (0.77; 12.34) *p* = 0.11	−0.07 (−0.29; 0.14) *p* = 0.49	−0.01 (−0.21; 0.20) *p* = 0.94	0.07 (−0.14; 0.28) *p* = 0.52

Time by park interaction with ‘park = control’ and underlined time moment as reference categories. *PA* = physical activity, *OR* = odds ratio, *CI* = confidence interval

^a^Gamma model with identity link, ^b^Hurdle model with part one = logistic regression and part two = gamma model with identity link. Bold results represent intervention effects

### Mean PA intensity level of park visitors

No evidence of intervention effects on the mean MET scores of the park visitors, either overall or per age category, was found.

## Discussion

To the best of our knowledge, this is the first European natural experiment that has examined the longer-term effects of park renewal on park-based PA among different age groups of park visitors. The findings show an increase in the total number of visitors engaged in sedentary and vigorous-intensity activities one and two years after the renewal, but no intervention effects for the total number of visitors walking or for the mean PA intensity levels of the visitors. Park improvements in this study were designed to promote park visitation by supporting a range of activities among all age groups, including vigorous PA (e.g., outdoor fitness equipment, playgrounds, multi-use sports cage), walking (e.g., wide concrete pathways accessible for individuals with disabilities, various benches for resting while walking) and sedentary activities (e.g., various benches and picnic tables with wheelchair cutouts). Age-specific results showed that, compared to the control park, there was an increase in the intervention park in the number of children and adolescents engaged in vigorous-intensity activities, and in adolescents walking one and two years after the renewal. Additionally, more adults were engaging in sedentary activities and walking, and more older adults were sedentary, walking and vigorously active in the intervention park. Similar to the Australian study by Paudel et al. [20], this study in Belgium showed that extensive park renewal can result in varied effects on activities of different intensities among different age groups.

In this study, no evidence was found for a longer-term intervention effect on the total number of visitors walking. This aligns with the post-test findings, which showed only a minimal increase in walking visitors following park renewal [25]. In contrast, a natural experiment conducted in Australia reported a sustained increase in the number of park visitors observed walking 12 months after park improvements. Notably, the intervention park in the Australian study included the addition of a large (12,800 m²) fenced, leash-free dog area as well as a walking track [23], which may have contributed to the differing outcomes. Previous studies have often grouped walking and more vigorous activities together under MVPA, whereas in our study we investigated these variables separately following the SOPARC protocol. This makes the comparison between our study and previous studies challenging. In our study, the increase in the total number of park visitors engaged in vigorous PA observed at post-test [25] was maintained one and two years later, indicating a stable long-term effect of the park renewal on vigorous PA. Similarly, the Australian study by Veitch et al. [23] also found increased numbers of visitors engaged in MVPA after the park renewal. Refurbishments in this study focussed on walking (i.e., large fenced dog area), but also on MVPA such as the installation of an all-abilities playground [23]. In contrast, two Australian studies in which the park renewals included fewer refurbishments [20] or the installation of an outdoor fitness equipment and multi-sports court [16], found no effects on the total number of visitors engaged in MVPA.

In our study, the number of children engaged in vigorous PA increased in the intervention park compared to the control park. In previous natural experiments in Australia, the number of children engaged in MVPA increased three months after the installation of a new fitness area [22], 12 and 24 months after the installation of landscaping and a nature-based play-scape [21], and eight and 24 months after an extensive park renewal [20]. However, more limited renovations in one of the parks in the study by Paudel et al. (2024) showed no change in the number of children engaged in MVPA [20]. In our study, six different playgrounds targeting children of different ages were installed in the intervention park. These playgrounds could have attracted more children to play in the park, which was also seen in other research in which the park renewal included the installation of a playground [20, 21]. Furthermore, in interviews conducted in the intervention park after the renewal, older adults mentioned that children also used the outdoor fitness equipment as a playground [29].

The odds of observing any adolescent walking in the intervention park were increased between pre-test and both follow-ups, compared to the control park. However, when at least one adolescent was observed walking in the park, the number of adolescents walking decreased between pre-test and follow-up one in the intervention park compared to the control park. Additionally between pre-test and follow-up two, when at least one adolescents was observed engaging in vigorous PA, the number of adolescents engaged in vigorous PA increased in the intervention park, compared to the control park. This may indicate that individual adolescents are more likely to walk in the intervention park after its renewal, such as when traveling from one place to another. Conversely, groups of adolescents may be less inclined to walk together in the park, preferring instead to engage in vigorous activities like football or basketball. This tendency could be attributed to features designed to appeal to groups of adolescents, such as the multi-use sports cage. Previous studies have reported no evidence of changes in the number of adolescents engaged in MVPA after park renovations. This lack of change may be due to the renovations not including enough features specifically designed to attract this age group [20–22].

More adults and older adults were observed walking in the intervention park at post-test and follow-up one, compared to pre-test and the control park. However, no intervention effect was found on the number of adults engaged in vigorous PA, whereas the number of older adults engaged in vigorous PA increased between pre-test and post-test and pre-test and follow-up two in the intervention park compared to the control park. Previous research showed mixed results, with both increases [21, 22] and decreases [20] in the number of (older) adults engaged in MVPA after park renewals. The renewed park in our study is still considered an urban pocket park, and might offer relatively few exploratory options compared to larger green spaces. Hence, it could be that after repeated visits, users of the renewed park may become familiar with all available routes, shifting their behaviour from exploratory wandering to targeted visits to preferred spots (i.e., activity or resting areas). Especially noteworthy in our study is the sustained increase in older adults engaged in vigorous activities. While previous research has reported increases in the number of older adults engaged in MVPA three, 12 [22] and 24 months [20] post intervention, they did not differentiate between walking and activities of higher intensity. Our findings show that park renewals have the potential to increase the number of older adults engaged in activities at a higher intensity. In this park renewal, attention was paid to the needs of older adults and the park renewal plans were co-designed with stakeholders from the adjacent elderly care facility including the director and physiotherapists. In a qualitative study conducted in the intervention park, older adults indicated that they liked the park additions promoting PA such as the outdoor fitness equipment [29].

The increase in the number of (older) adults observed sedentary in the intervention park relative to the control park could potentially be attributed to newly installed features promoting sedentary behaviour, such as various benches and picnic tables accessible to people with disabilities. Furthermore, there was a growing trend in the number of visitors observed sedentary after the park renewal, with a significant increase among both adults and older adults. This suggests that intervention effects on the number of park visitors observed sedentary were not only persistent over time but also continued to grow several years after completion of the park renewal. These results should not be considered detrimental, as visiting parks offers numerous benefits for the mental and social wellbeing of visitors. Additionally, the park visitors observed sedentary during the observations may have been physically active while traveling to the park or during other parts of their park visit that were not captured during the observations. Previous research primarily focussed on visitors’ MVPA and did not investigate changes in the number of visitors observed sedentary [19–21, 23]. However, Paudel et al. [20] suggested that the decrease or lack of change in the mean PA intensity levels of the visitors in their study, could be attributed to an increase in the number of sedentary visitors, as they found an increase in the number of people engaging in vigorous PA.

Consistent with findings at post-test [25], no evidence was found for a change in the mean PA activity levels of the park visitors between pre-test and follow-up one or two. This aligns with previous research [20, 35]. In our study, park improvements aimed to promote a range of activities among different age groups, including vigorous PA, walking and sedentary activities. The observed increases in the total number of park visitors observed sedentary and vigorously active may have impacted the lack of overall change in the mean PA activity level of the visitors. As park visitation provides many physical, mental and social health benefits, regardless of the activities performed by the visitor [4], the lack of evidence for a change in MET scores should not be considered a deterrent for park renewal. Rather, the increase in the total number of sedentary and vigorously active park visitors can be considered successful results of the park renewal as more people will be benefiting from the health promoting park environment [26].

At both the post-test and follow-up one, Covid-19 restrictions were in effect in Belgium, particularly in care institutions like elderly care facilities. While these restrictions applied equally to both parks at all times, they may have been a barrier for park visitation, especially among residents of the nearby elderly care facility and their visitors. The restrictions were stricter at post-test than at follow-up one and were completely lifted by follow-up two, which could explain the increasing trend in the number of (older adult) visitors observed sedentary. Furthermore, previous research has indicated that green space interventions, such as park renewals, may take several years to result in new visiting behaviours [36]. However, this study showed that, except for walking, the effects of park renewal found immediately after completion are maintained one and two years later. Hence, the benefits of urban park renewal were in general maintained over time. This study provides valuable evidence for urban planners and decision-makers, and may potentially guide decisions on allocating funds for green space investments, as the results highlight the enduring impact of park renewal.

## Strengths and limitations

This study has several strengths. It is the first European natural experiment focusing on the longer-term effects of park renewal on park-based PA, incorporating two follow-up periods, 18 and 30 months post intervention. The SOPARC tool, recognized for its standardization, validity, and reliability, was used to collect objective data on park visitation. This tool is widely used in park research, which facilitates cross-study comparison and enhances generalizability of findings [30]. Furthermore, all observations were conducted by trained researchers and, to minimize contextual or weather-related variability, all observations in both the intervention and control park were performed on the same days and at the same times. This study also investigated the effects of park renewal on park-based PA by age group, which had previously only been performed in Australian research [20–22]. Nevertheless, the study is not without limitations. A delay in the park renewal resulted in a large time lag between pre-test observations in 2014 and post-test and follow-up observations, conducted from 2020 to 2022 by different researchers. To mitigate this, all researchers were thoroughly trained using the SOPARC protocol and instructional videos, and excellent interrater reliability between raters from the same wave was established prior to each data collection. Furthermore, the post-test and first follow-up observations coincided with the Covid-19 pandemic, during which restrictions were in place. However, these restrictions affected both the intervention and control park. In addition, there were changes in the accessible size of the intervention park, due to both the renewal and pandemic-related restrictions, but these were accounted for in the statistical analysis. Furthermore, the study only included one control site and one pre-test measurement period. Including multiple matched control sites and pre-test measurements would enhance the internal validity of this type of studies [24, 37]. Lastly, because park renewals often spanned multiple target areas, it was not feasible to attribute the observed effects to any single feature or to disentangle their individual impacts from other concurrent modifications. Future research would benefit from isolating and evaluating the influence of specific changes, such as the installation of seating or outdoor fitness equipment, on visitors’ physical activity levels.

## Conclusion

This study showed longer-term intervention effects of an urban park renewal on the total number of visitors observed sedentary and vigorously active. However, no significant changes were observed in the total number of visitors walking or the mean PA intensity levels of the visitors. Because public parks also provide valuable opportunities for promoting mental and social health, the increased number of visitors observed sedentary should not be considered detrimental. Varying results were found according to age group of the park visitors. Notably, all intervention effects, except the increase in the number of (older) adults observed walking, were maintained two years after the completion of the renewal. Hence, the benefits of urban park renewal seem to be maintained over time.

## Supplementary Information

Supplementary Material 1.

Supplementary Material 2.

Supplementary Material 3.

Supplementary Material 4.

## Data Availability

The datasets generated and/or analysed during the current study are available in the Open Science Framework repository, at [https:/osf.io/2z7sg].
