# Supplementary material for: Evaluating the impact of park renovation on park-based physical activity: a natural experiment in Belgium with two years of follow-up
Source: Int J Behav Nutr Phys Act. 2025 Dec 5;22:154. doi: 10.1186/s12966-025-01846-0 (PMC12681091; doi:10.1186/s12966-025-01846-0)
Supplement: Supplementary file 1 — Supplementary Material 1. [file 12966_2025_1846_MOESM1_ESM.docx]

Supplementary File 3

The graphs of the post hoc analyses for non-significant omnibus likelihood ratio tests that are presented in Supplementary file 3 should be interpret with caution due to the higher likelihood of type II errors.

# Sedentary park visitors

## Children observed sedentary


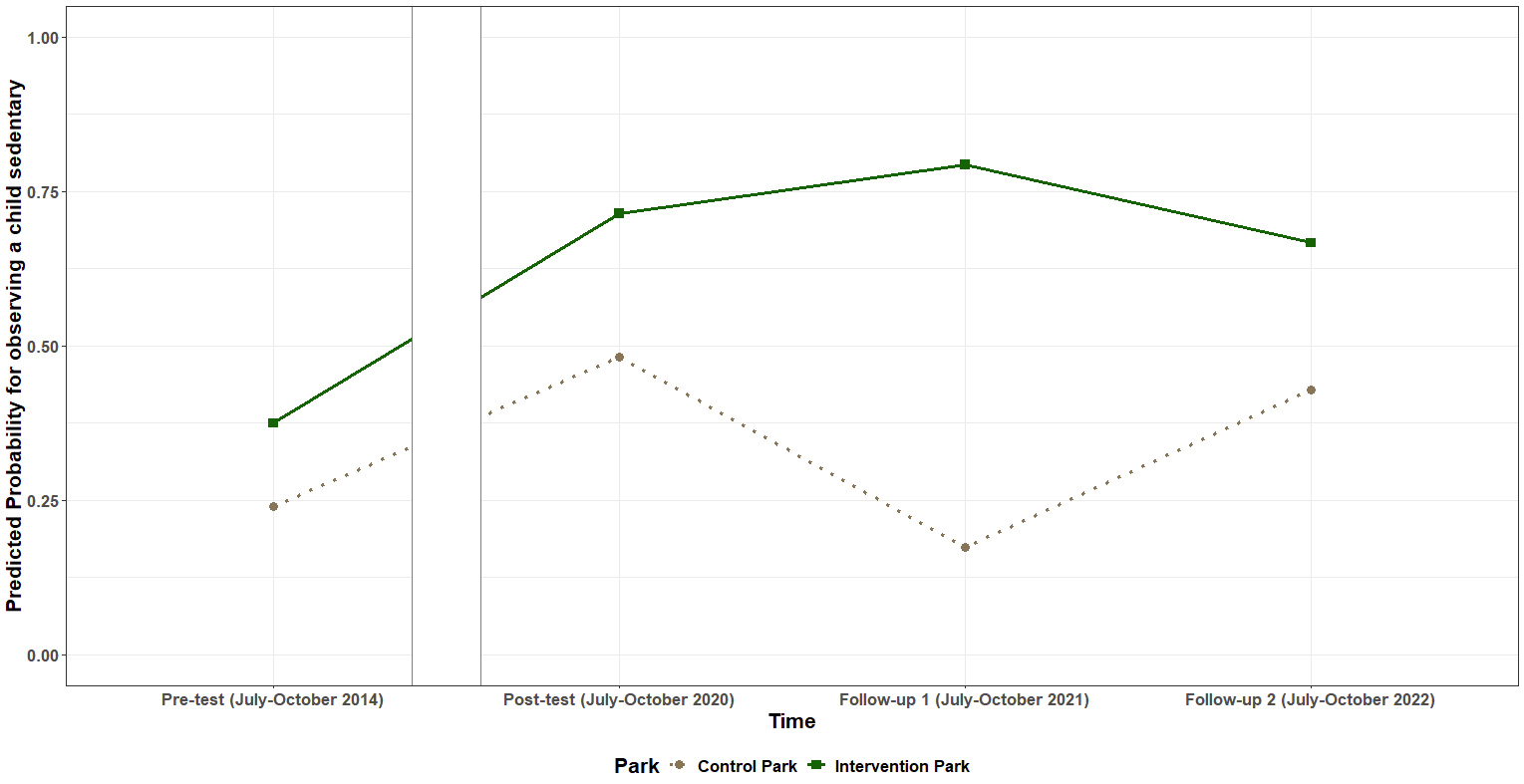


Figure 1: Estimated probability for observing a child sedentary per timepoint in the intervention and control park.


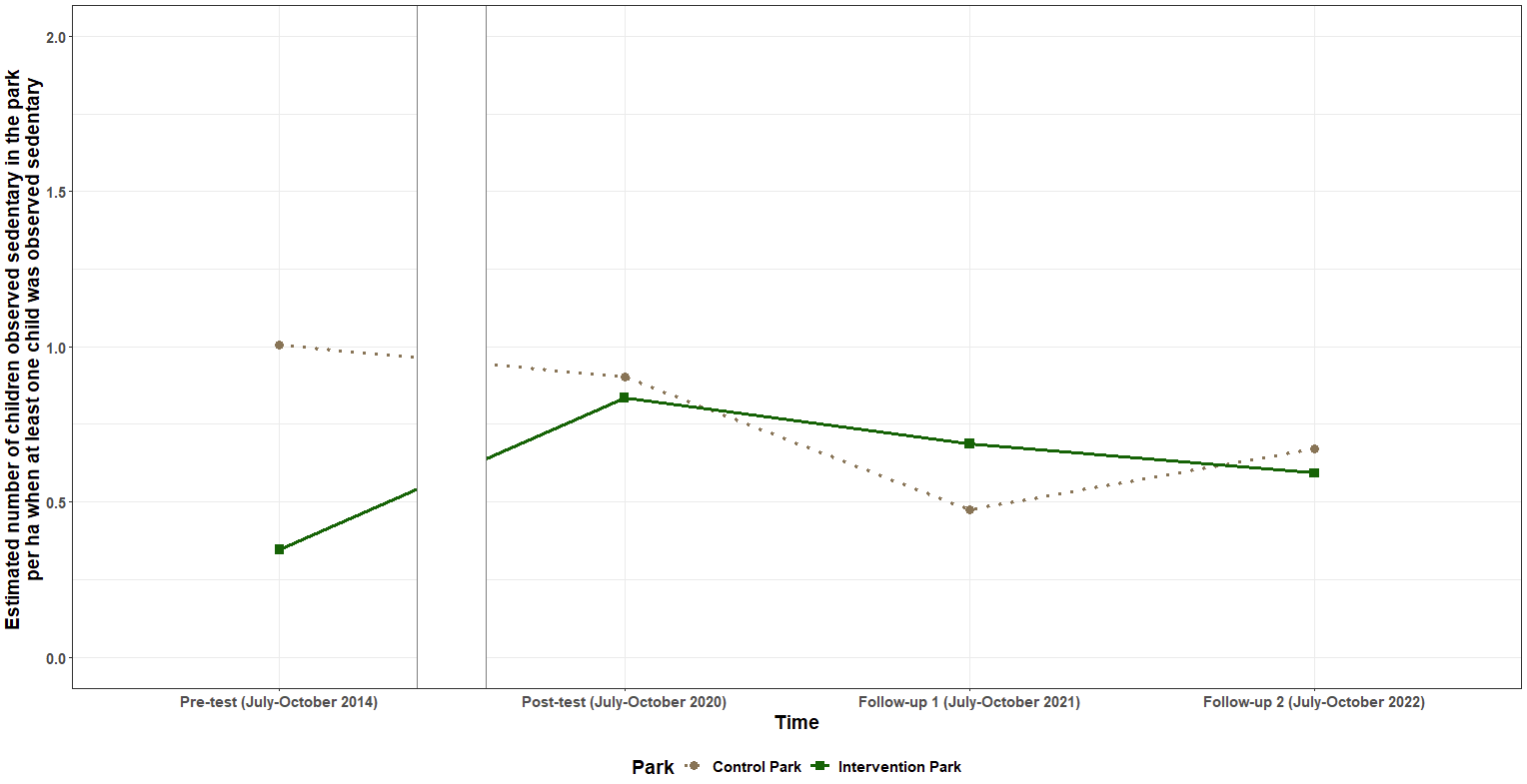


Figure 2: Estimated number of children/ha observed sedentary per timepoint for the intervention and control park when at least one child was observed sedentary.

## Adolescents observed sedentary


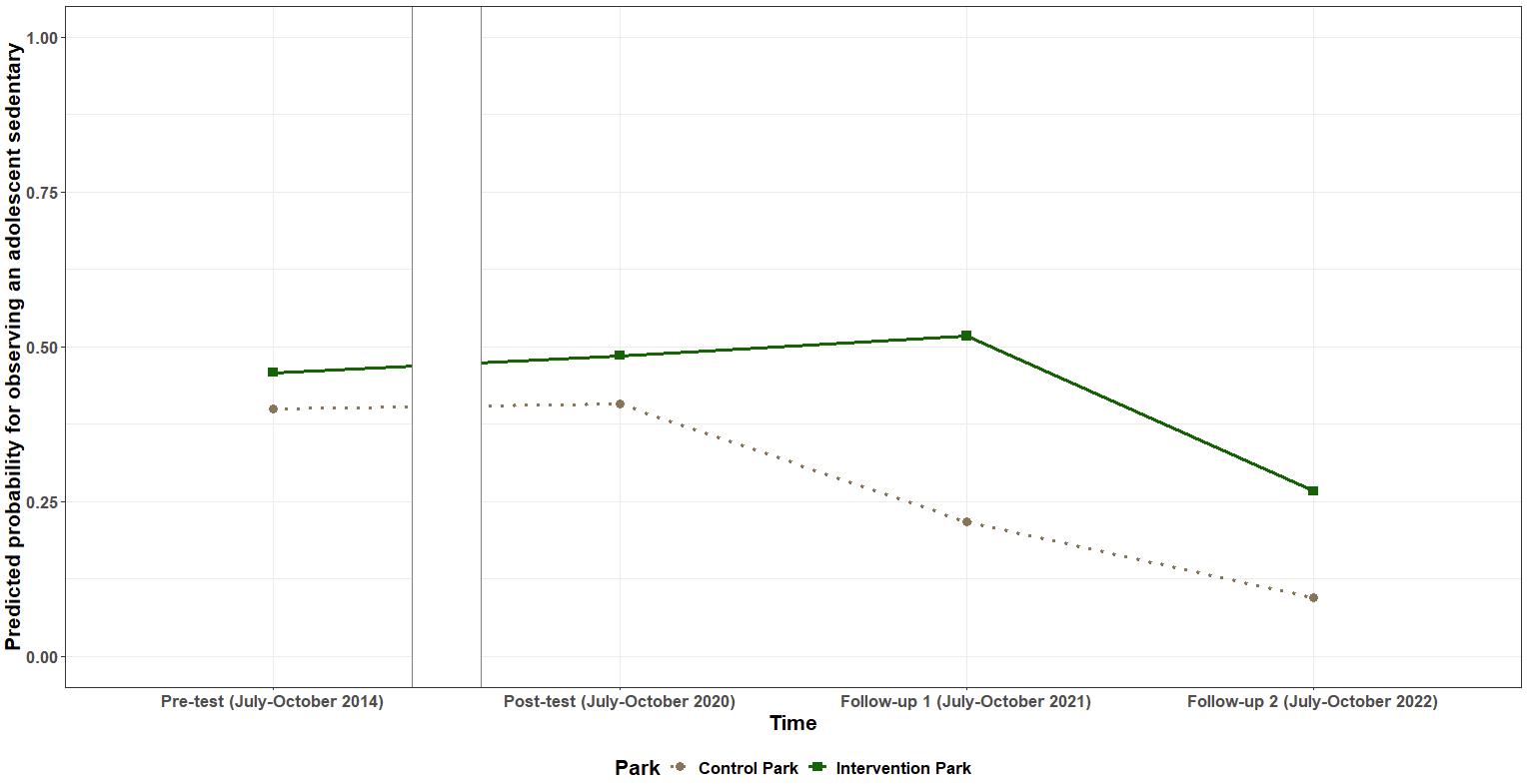


Figure 3: Estimated probability for observing an adolescent sedentary per timepoint in the intervention and control park.


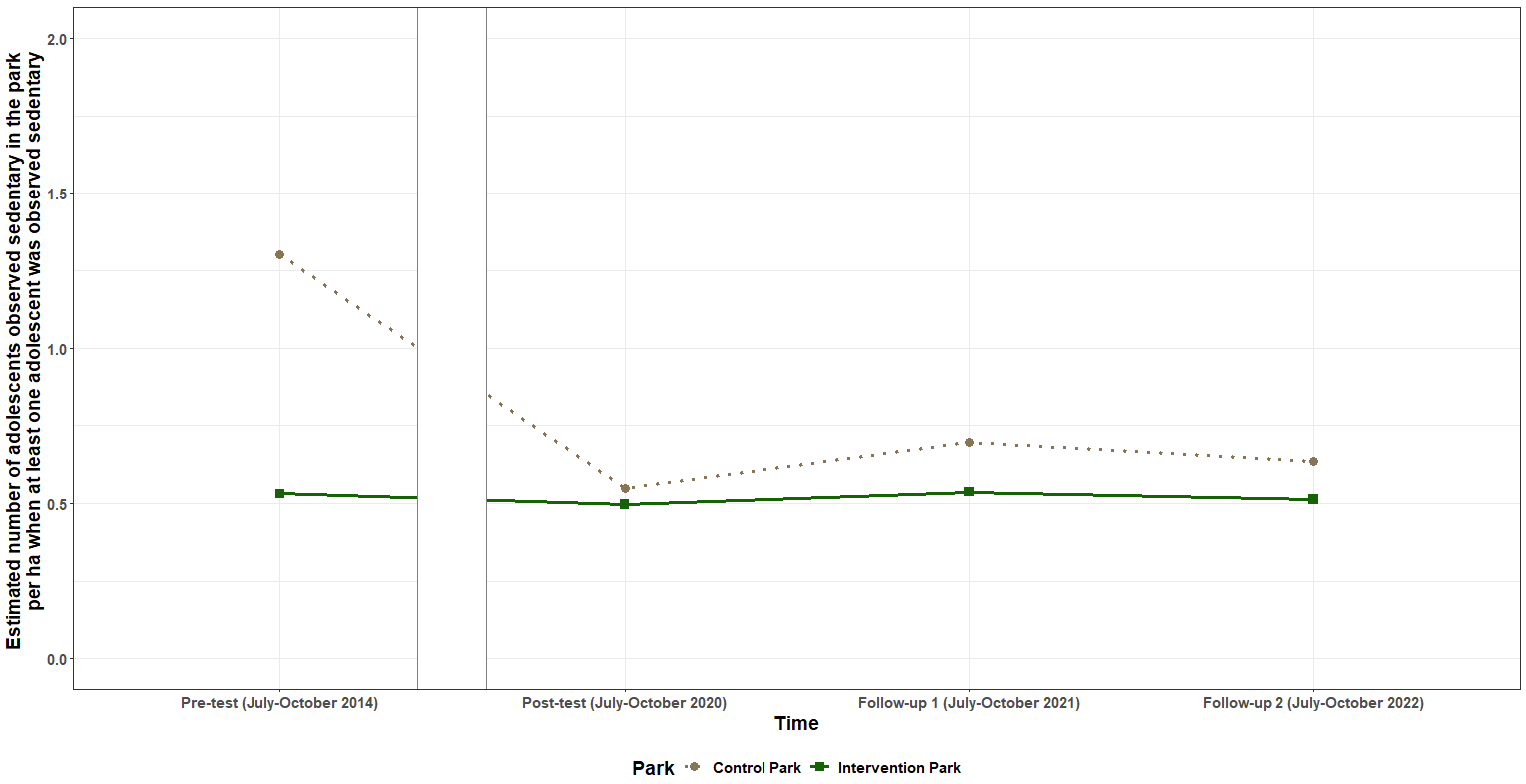


Figure 4: Estimated number of adolescents/ha observed sedentary per timepoint for the intervention and control park when at least one adolescent was observed sedentary.

## Adults observed sedentary


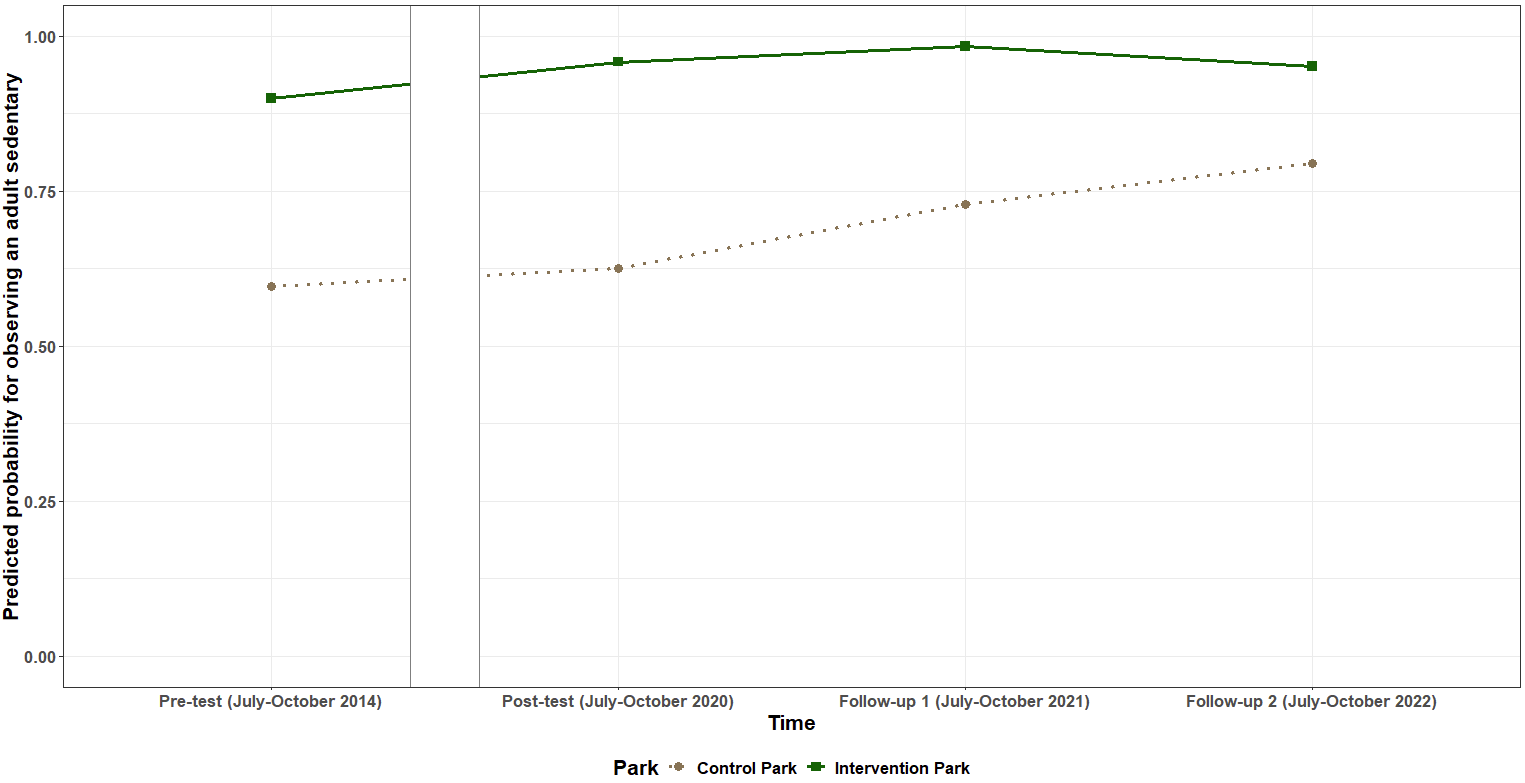


Figure 5: Estimated probability for observing an adult sedentary per timepoint in the intervention and control park.

## Older adults observed sedentary


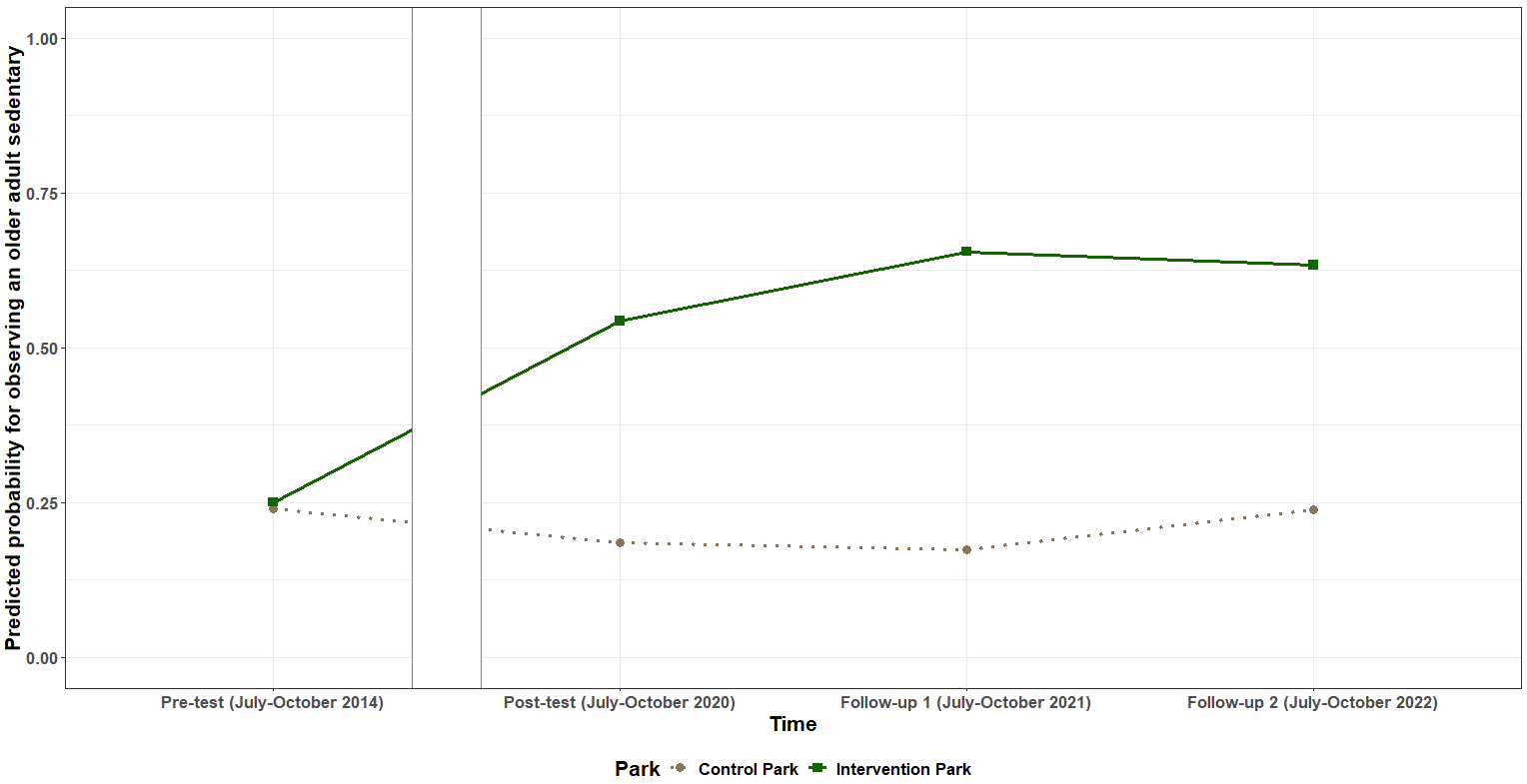


Figure 6: Estimated probability for observing an older adult sedentary per timepoint in the intervention and control park.

# Park visitors observed walking

## Total number of park visitors walking


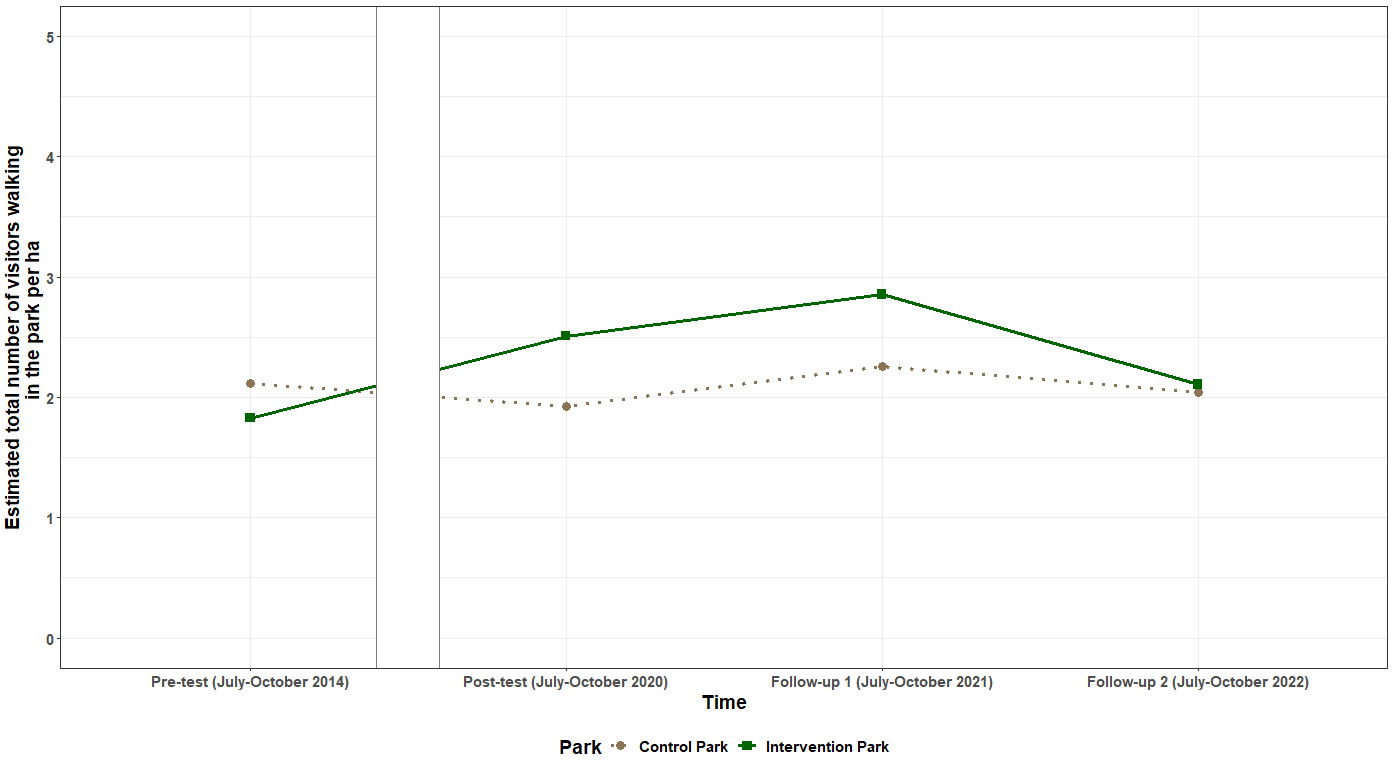


Figure 7: Estimated total number of visitors/ha walking per timepoint for the intervention and control park.

## Children walking


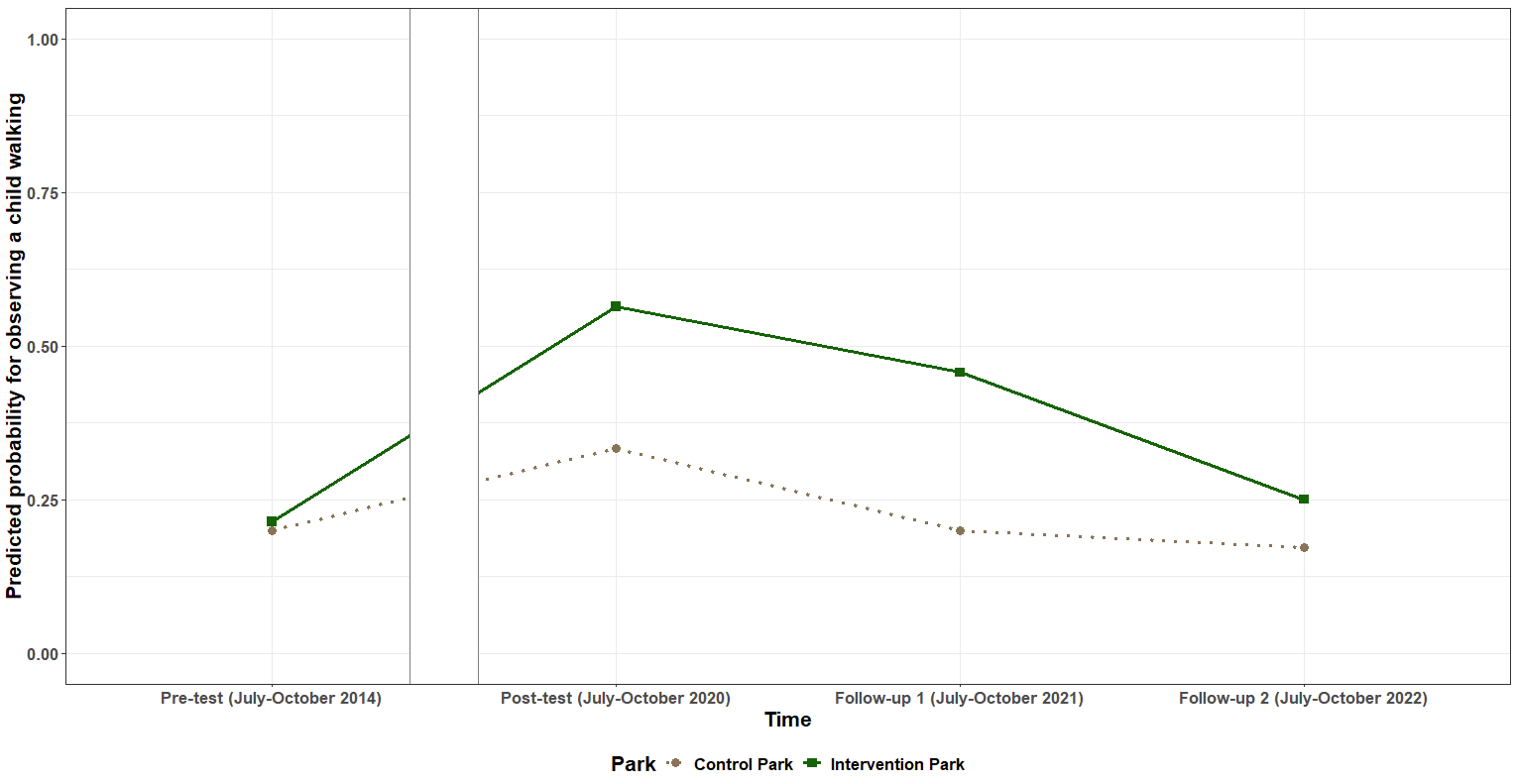


Figure 8: Estimated probability for observing a child walking per timepoint in the intervention and control park.


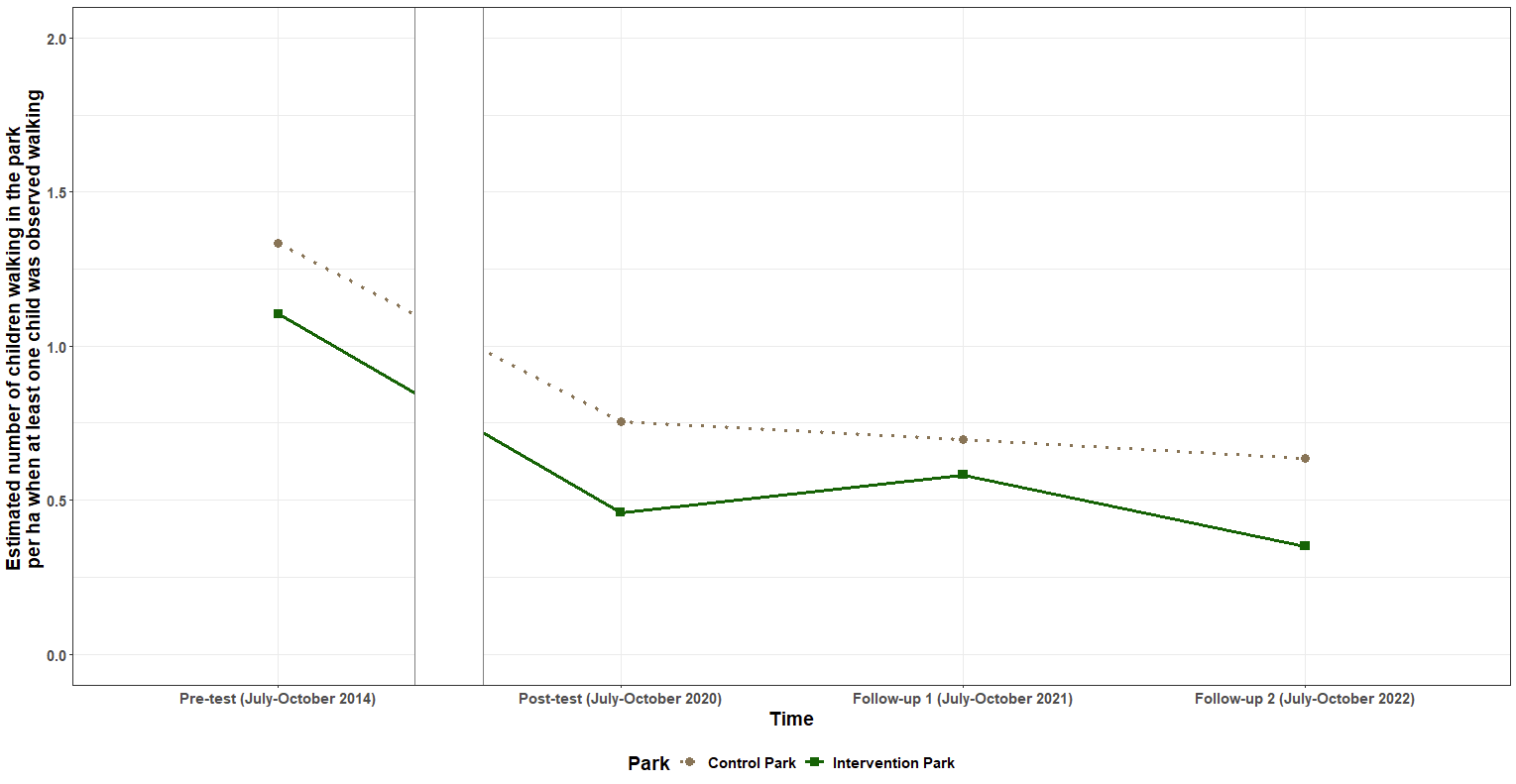


Figure 9: Estimated number of children/ha walking per timepoint for the intervention and control park when at least one child was observed walking.

## Adults walking


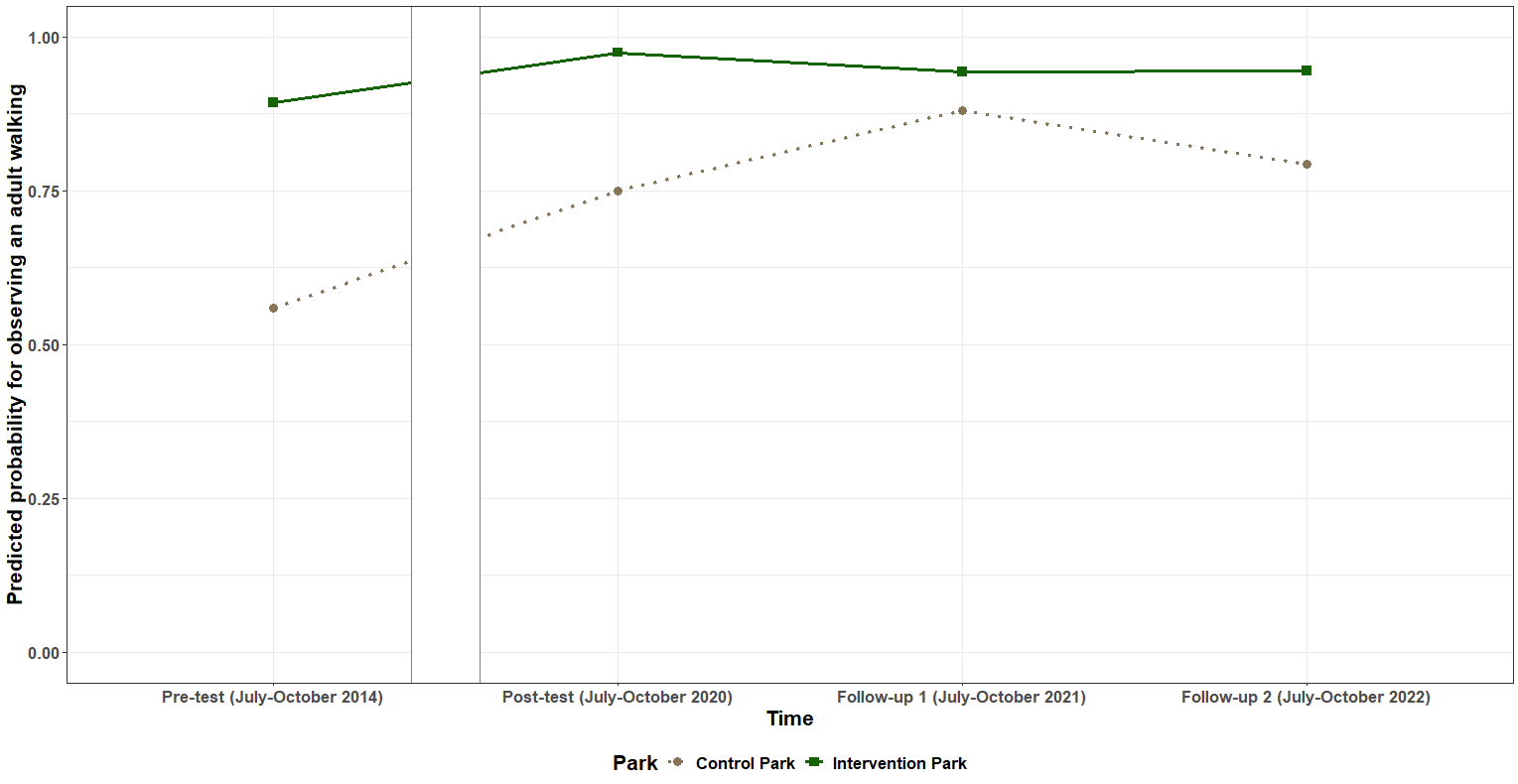


Figure 10: Estimated probability for observing an adult walking per timepoint in the intervention and control park.

## Older adults walking


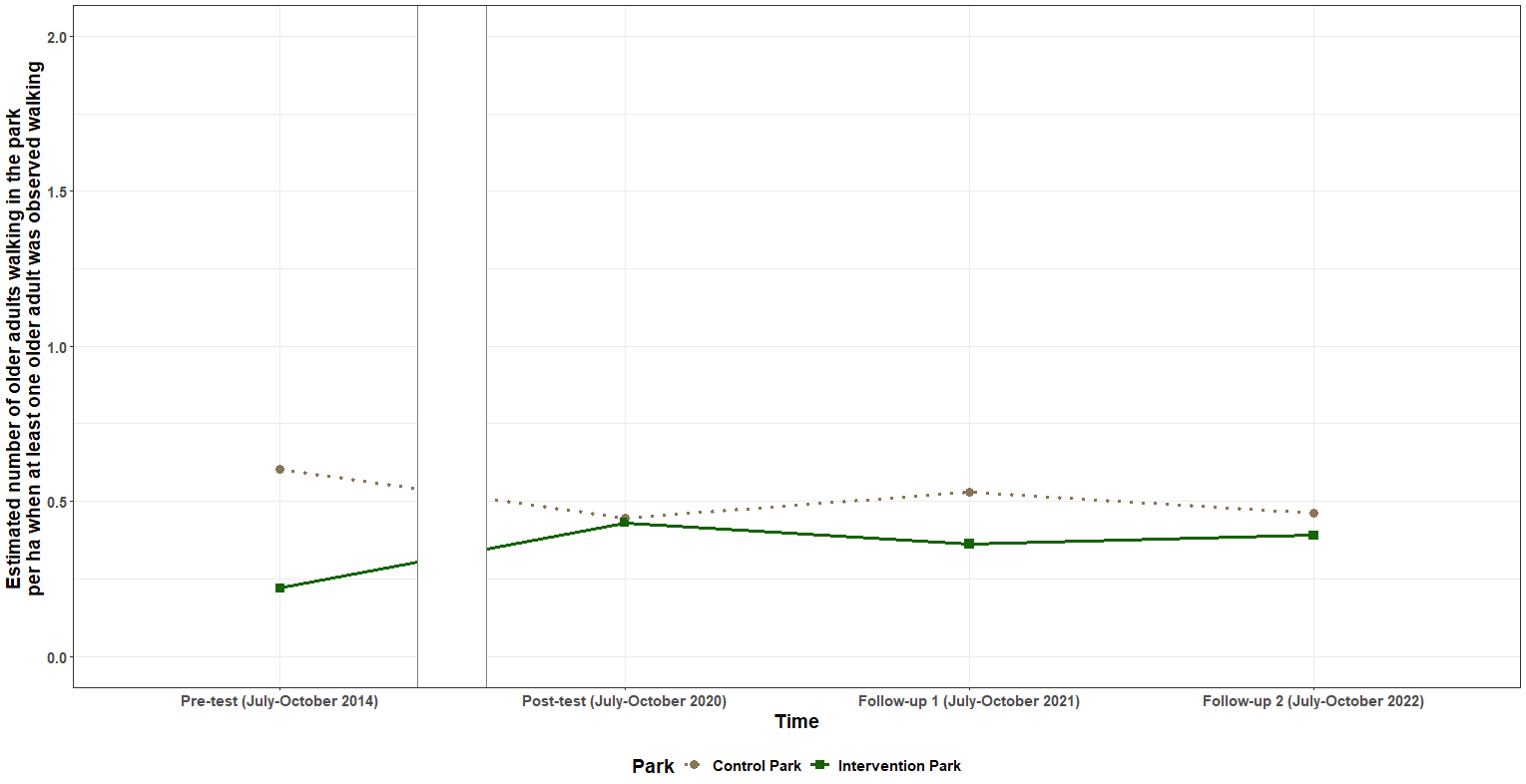


Figure 11: Estimated number of older adults/ha walking per timepoint for the intervention and control park when at least one older adult was observed walking.

# Park visitors engaged in vigorous-intensity activities

## Children engaged in vigorous PA


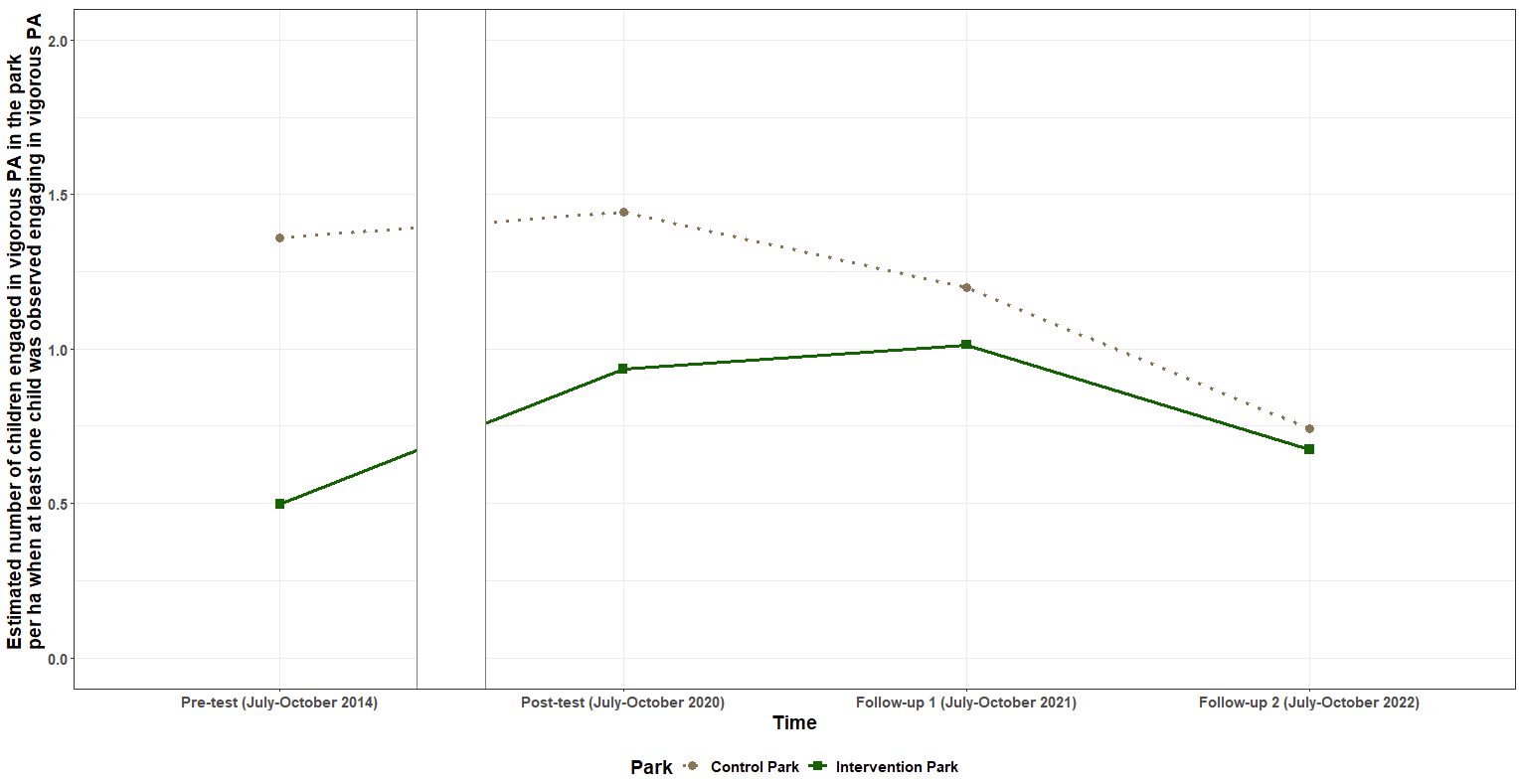


Figure 12: Estimated number of children/ha engaged in vigorous PA per timepoint for the intervention and control park when at least one child was observed engaging in vigorous PA.

## Adolescents engaged in vigorous PA


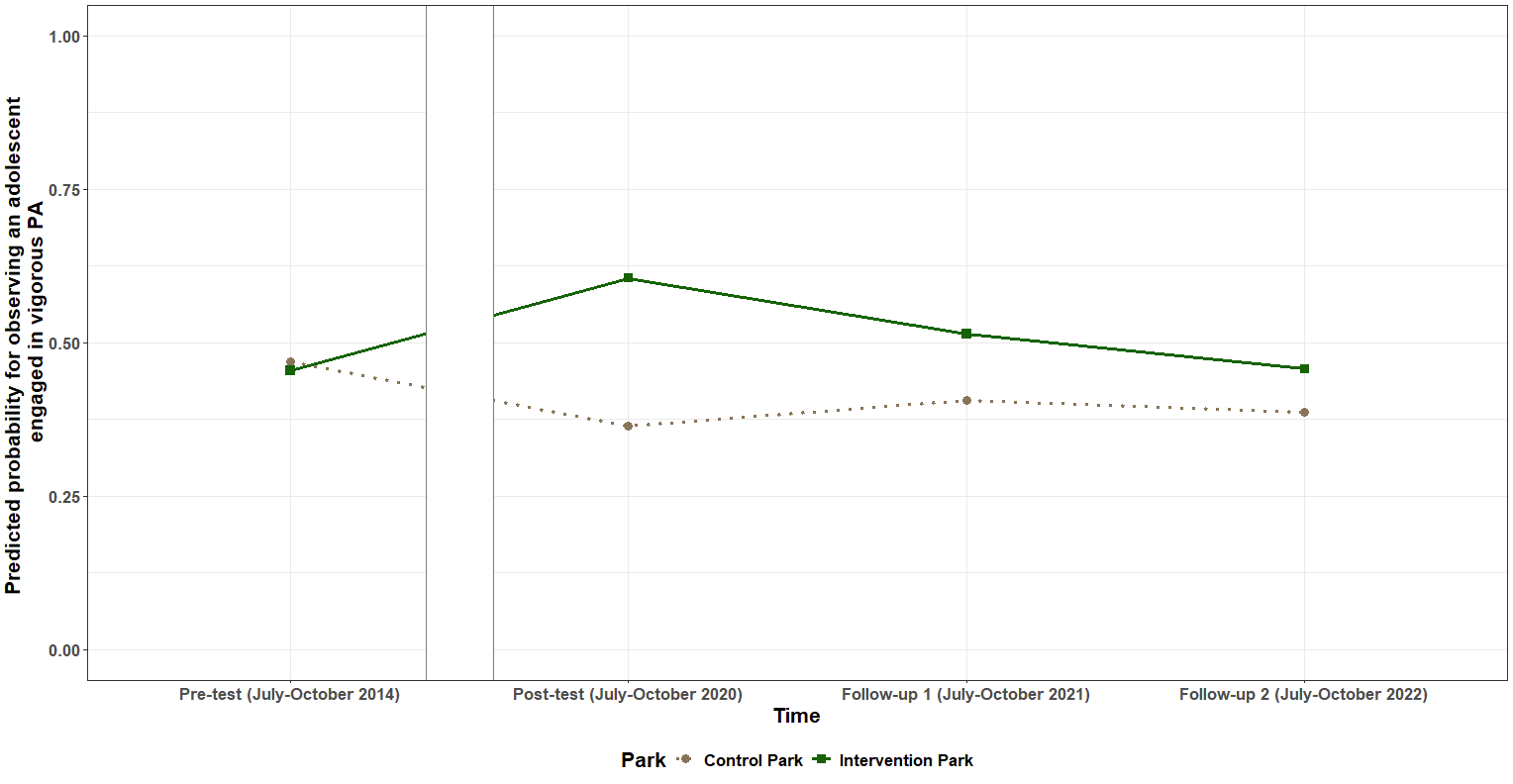


Figure 13: Estimated probability for observing an adolescent engaged in vigorous PA per timepoint in the intervention and control park.

## Adults engaged in vigorous PA


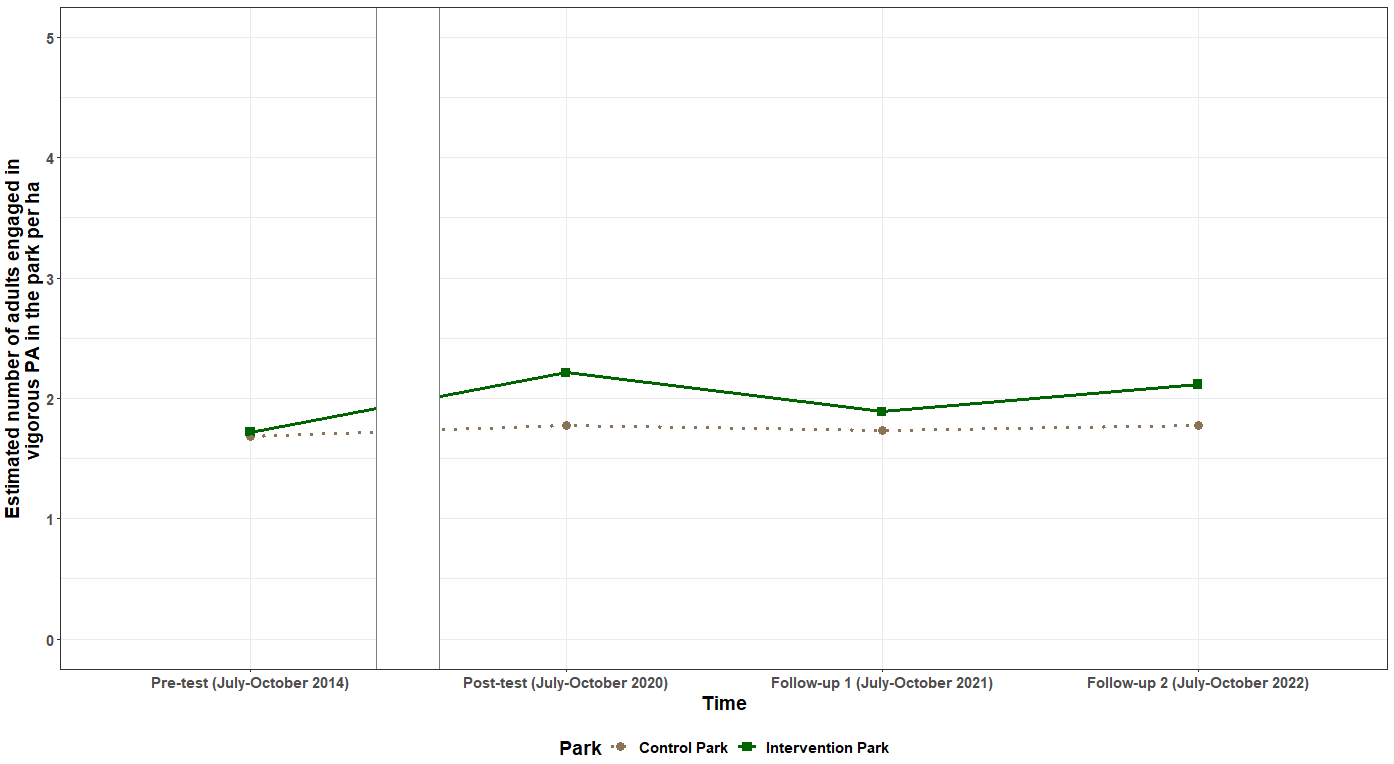


Figure 14: Estimated number of adults/ha engaged in vigorous PA per timepoint for the intervention and control park.

## Older adults engaged in vigorous PA


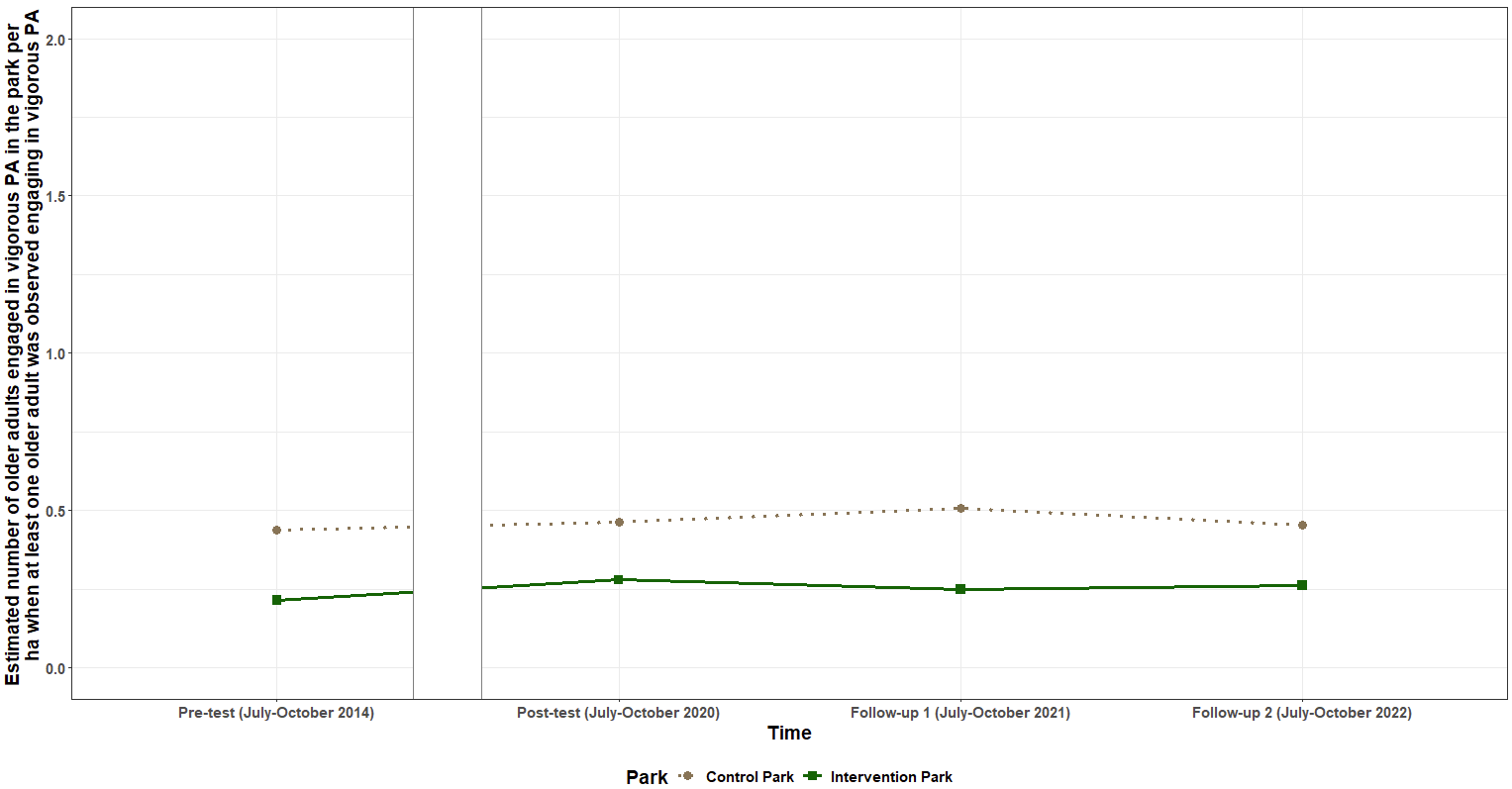


Figure 15: Estimated number of older adults/ha engaged in vigorous PA per timepoint for the intervention and control park when at least one older adult was observed engaging in vigorous PA.

# Mean PA intensity level of the park visitors


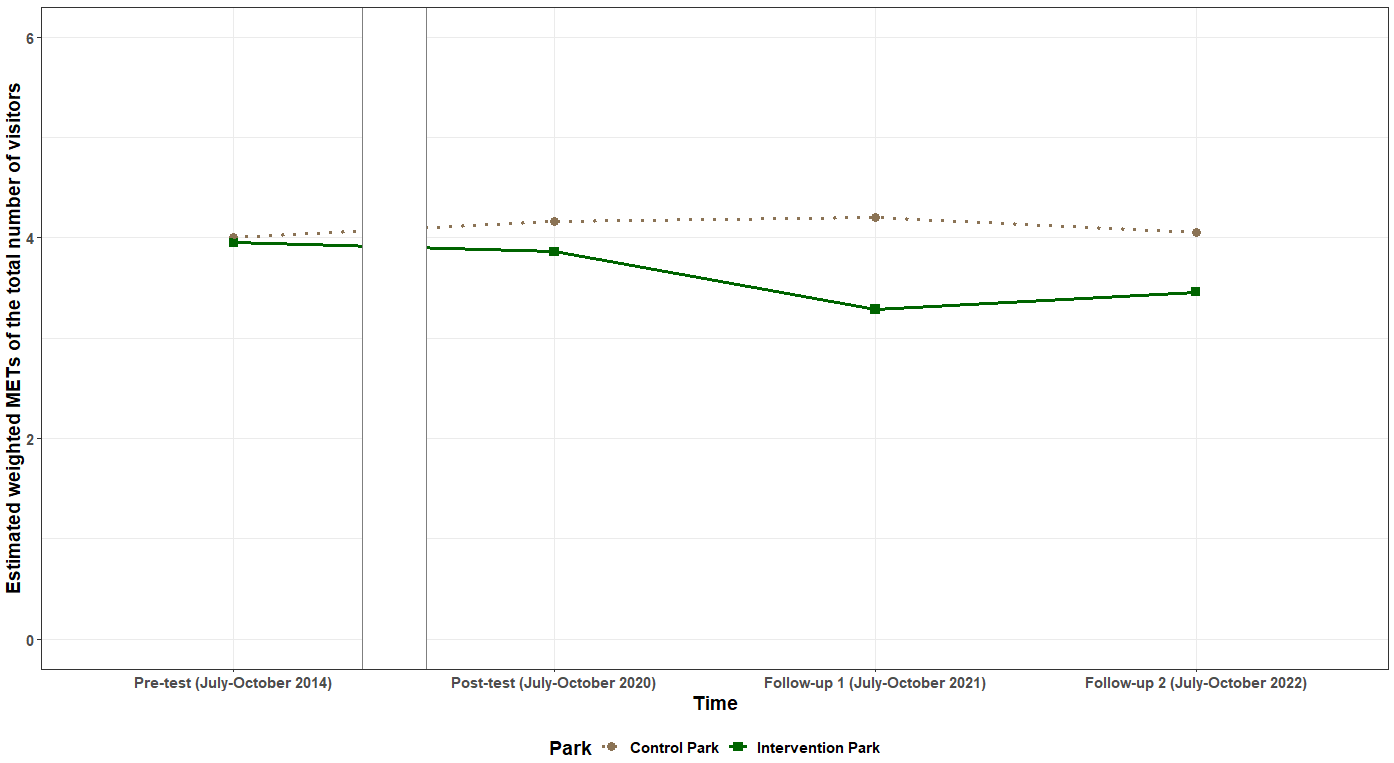


Figure 16: Estimated weighted MET scores of the total number of park visitors per timepoint for the intervention and control park.


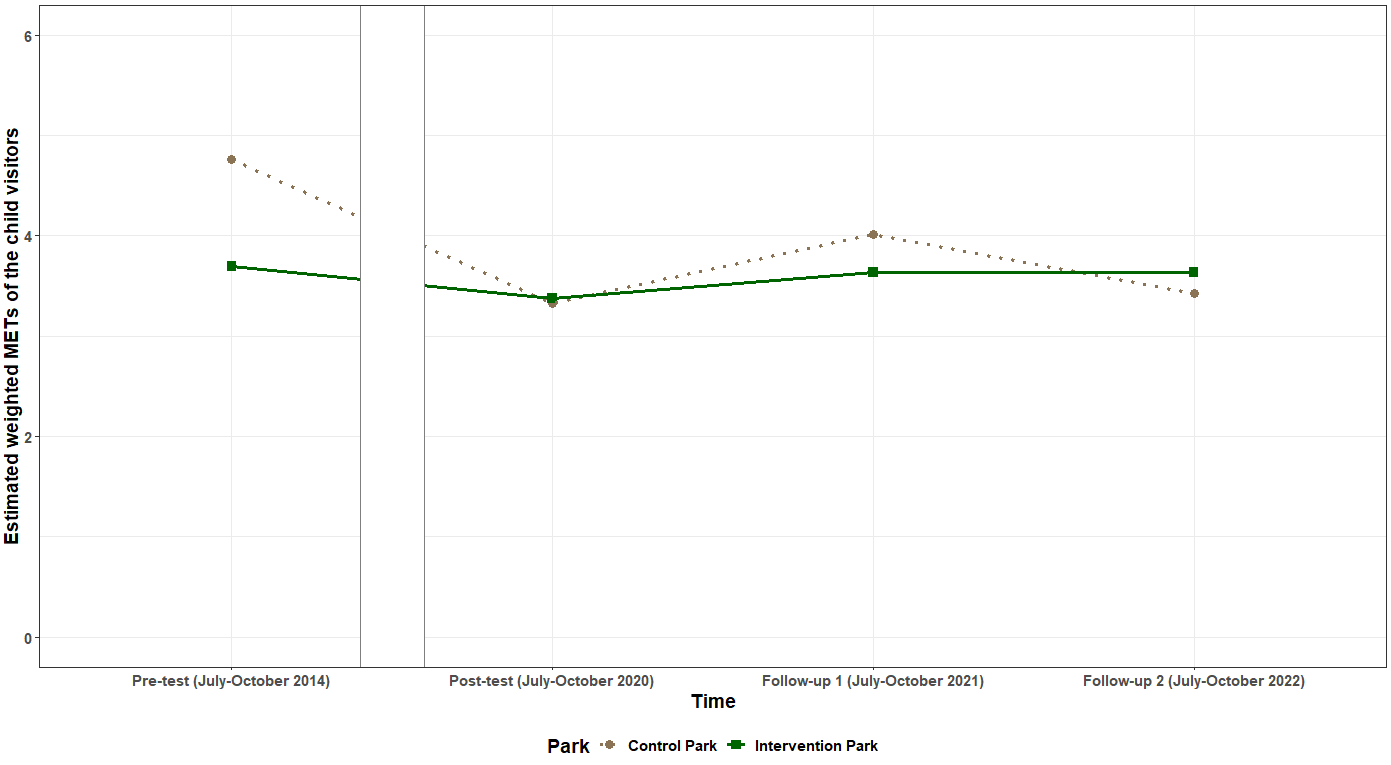


Figure 17: Estimated weighted MET scores of the children per timepoint for the intervention and control park.


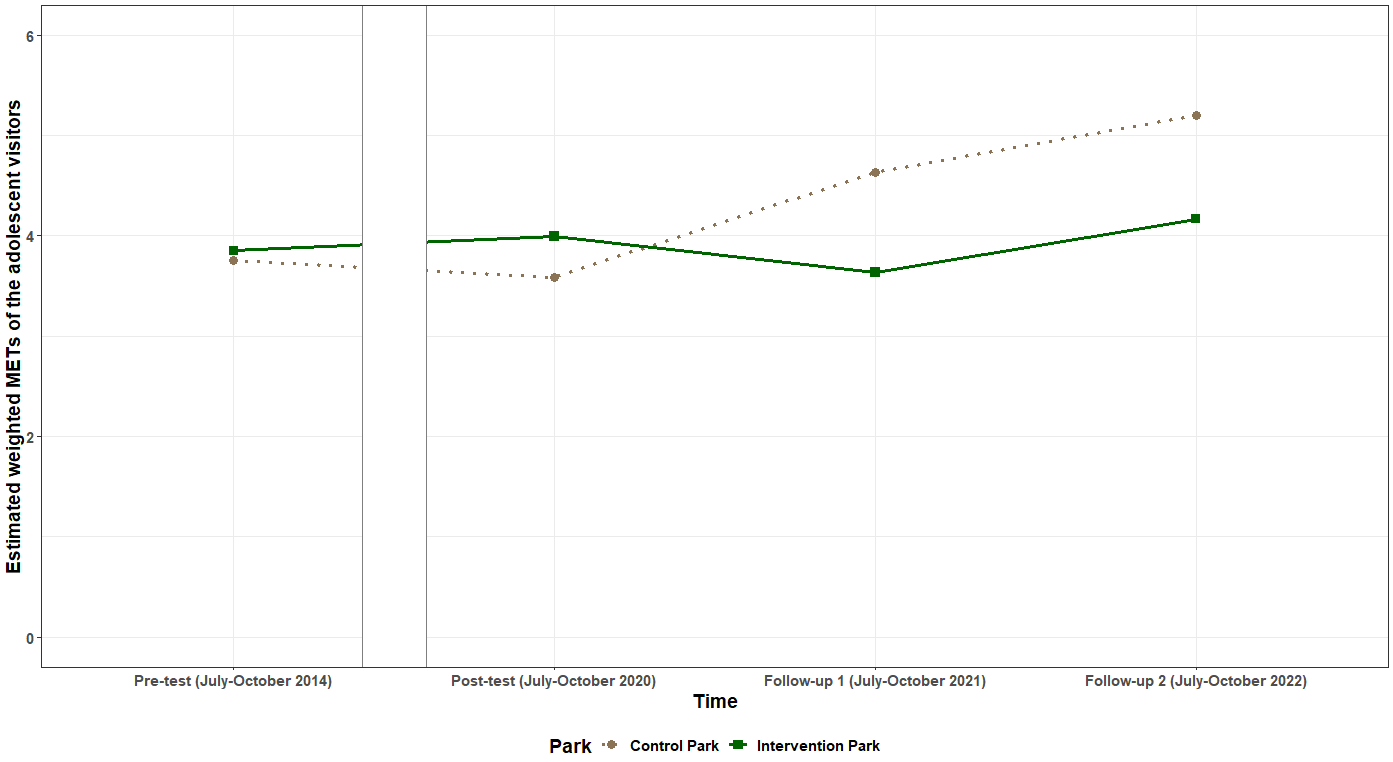


Figure 18: Estimated weighted MET scores of the adolescents per timepoint for the intervention and control park.


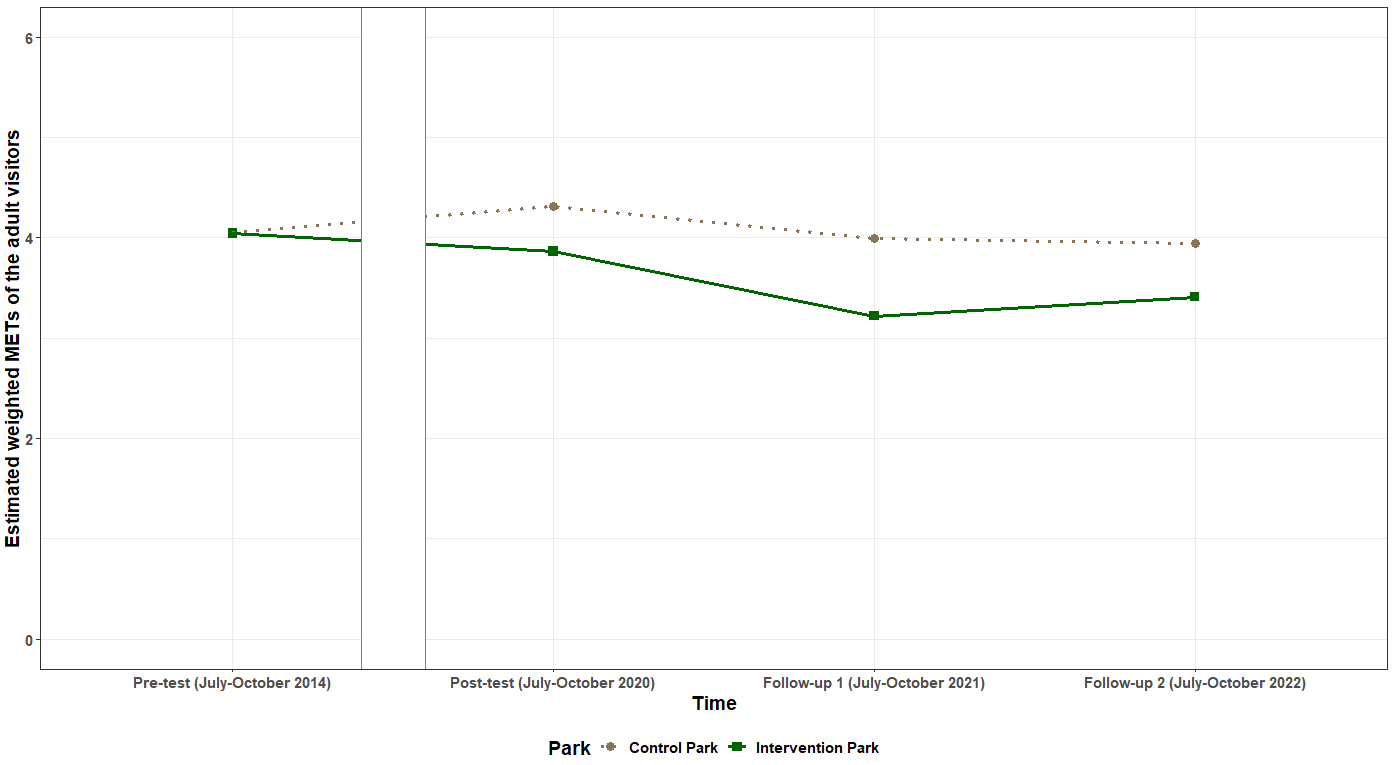


Figure 19: Estimated weighted MET scores of the adults per timepoint for the intervention and control park.


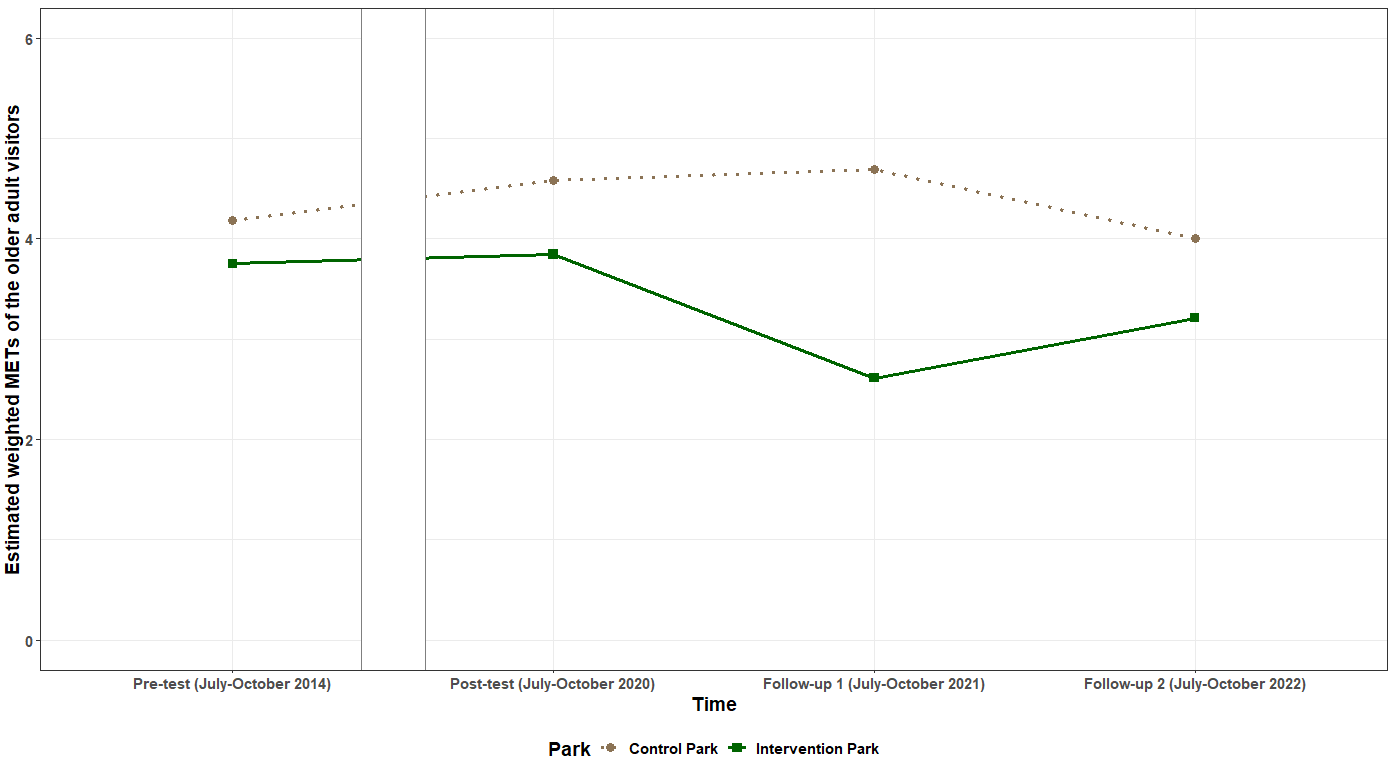


Figure 19: Estimated weighted MET scores of the older adults per timepoint for the intervention and control park.
