# Supplementary material for: Evaluating the impact of park renovation on park-based physical activity: a natural experiment in Belgium with two years of follow-up
Source: Int J Behav Nutr Phys Act. 2025 Dec 5;22:154. doi: 10.1186/s12966-025-01846-0 (PMC12681091; doi:10.1186/s12966-025-01846-0)
Supplement: Supplementary file 2 — Supplementary Material 2. [file 12966_2025_1846_MOESM2_ESM.docx]

Supplementary file 1

Table 1: Omnibus likelihood ratio test scores for each dependent variable.

|  | **Regular Model** | **Zero Adjusted Model** | | |
| --- | --- | --- | --- | --- |
|  | **Gamma Identity Model** | | **Logistic Model** | **Gamma Identity Model** |
|  | Time x Park  χ² (df) p-value | | Time x Park  χ² (df) p-value | Time x Park  χ² (df) p-value |
| **Park visitors observed sedentary** | | | | |
| Total | **31.85 (3) p<0.001** | |  |  |
| Children |  | | 7.53 (3) p=0.06 | 4.15 (3) p=0.25 |
| Adolescents |  | | 2.73 (3) p=0.44 | 4.27 (3) p=0.23 |
| Adults |  | | 0.88 (3) p=0.83 | **22.03 (3) p<0.001** |
| Older adults |  | | 5.87 (3) p=0.12 | **11.77 (3) p=0.008** |
| **Park visitors observed walking** | | | | |
| Total | 6.00 (3) p=0.11 | |  |  |
| Children |  | | 1.88 (3) p=0.60 | 0.24 (3) p=0.97 |
| Adolescents |  | | **10.68 (3) p=0.01** | **12.40 (3) p=0.006** |
| Adults |  | | 1.59 (3) p=0.66 | **13.57 (3) p=0.004** |
| Older adults |  | | **13.93 (3) p=0.003** | 3.43 (3) p=0.33) |
| **Park visitors observed engaged in vigorous PA** | | | | |
| Total | **14.02 (3) p=0.003** | |  |  |
| Children |  | | **13.94 (3) p=0.003** | 2.32 (3) p=0.51 |
| Adolescents |  | | 2.35 (3) p=0.50 | **8.95 (3) p=0.03** |
| Adults | 3.71 (3) p=0.29 | |  |  |
| Older adults |  | | **7.92 (3) p=0.05** | 0.61 (3) p=0.90 |
| **Mean PA intensity level** | | | | |
| Total | 6.18 (3) p=0.10 | |  |  |
| Children | 3.35 (3) p=0.34 | |  |  |
| Adolescents | 5.95 (3) p=0.11 | |  |  |
| Adults | 3.00 (3) p=0.39 | |  |  |
| Older adults | 7.00 (3) p=0.07 | |  |  |

Notes. Bold results represent intervention effects which are discussed further in the manuscript.
