# Supplementary material for: Evaluating the impact of park renovation on park-based physical activity: a natural experiment in Belgium with two years of follow-up
Source: Int J Behav Nutr Phys Act. 2025 Dec 5;22:154. doi: 10.1186/s12966-025-01846-0 (PMC12681091; doi:10.1186/s12966-025-01846-0)
Supplement: Supplementary file 3 — Supplementary Material 3. [file 12966_2025_1846_MOESM3_ESM.docx]

Supplementary file 2

The results of the post hoc analyses for non-significant omnibus likelihood ratio tests that are presented in Supplementary file 2 should be interpret with caution due to the higher likelihood of type II errors.

# Intervention effects for the number of park visitors observed sedentary

Table 1: Intervention effects for the number of park visitors observed sedentary.

|  | Intervention effect for the odds of observing any visitor of the specified category sedentary | | | Intervention effects when at least one visitor of the specified category is observed sedentary | | |
| --- | --- | --- | --- | --- | --- | --- |
|  | **Pre-test – Post-test**  Time x Park OR (95% CI)  p value | **Pre-test – Follow-up 1**  Time x Park OR (95% CI)  p value | **Pre-test – Follow-up 2**  Time x Park OR (95% CI)  p value | **Pre-test – Post-test**  Time x Park beta (95% CI)  p value | **Pre-test – Follow-up 1**  Time x Park beta (95% CI)  p value | **Pre-test – Follow-up 2**  Time x Park beta (95% CI)  p value |
| Children^b^ | 1.42 (0.28; 7.18)  p=0.67 | **9.58 (1.48; 62.15)**  **p=0.02** | 1.40 (0.26; 7.59)  p=0.69 | 0.59 (-0.35; 1.53)  p=0.22 | 0.87 (-0.05; 1.79)  p=0.06 | 0.58 (-0.32; 1.48)  p=0.20 |
| Adolescents^b^ | 1.08 (0.24; 4.95) p=0.92 | 3.04 (0.57; 16.18) p=0.19 | 2.72 (0.36; 20.42) p=0.33 | 0.72 (-0.06; 1.50) p=0.07 | 0.61 (-0.28; 1.50)  p=0.18 | 0.65 (-0.40; 1.70)  p=0.22 |
| Adults^b^ | 2.26 (0.20; 25.02) p=0.51 | 3.59 (0.12; 104.34)  p=0.46 | 0.83 (0.07; 10.25) p=0.88 |  |  |  |
| Older adults^b^ | 4.95 (0.86; 28.64) p=0.07 | **8.55 (1.34; 54.71) p=0.02** | 5.24 (0.86; 31.82) p=0.07 |  |  |  |
|  | **Post-test – Follow-up 1**  Time x Park OR (95% CI)  p value | **Post-test – Follow-up 2**  Time x Park OR (95% CI)  p value | **Follow-up 1 – Follow-up 2**  Time x Park OR (95% CI)  p value | **Post-test – Follow-up 1**  Time x Park beta (95% CI)  p value | **Post-test – Follow-up 2**  Time x Park beta (95% CI)  p value | **Follow-up 1 – Follow-up 2**  Time x Park beta (95% CI)  p value |
| Children^b^ | **6.76 (1.17; 39.09) p=0.03** | 0.99 (0.21; 4.71) p=0.99 | **0.15 (0.02; 0.90) p=0.04** | 0.28 (-0  46; 1.02) p=0.46 | -0.01 (-0.73; 0.71) p=0.98 | -0.29 (-0.98; 0.40) p=0.41 |
| Adolescents^b^ | 2.81 (0.57; 13.82) p=0.20 | 2.51 (0.36; 17.69) p=0.35 | 0.90 (0.11; 7.11) p=0.92 | -0.11 (-0.75; 0.53) p=0.73 | -0.07 (-0.91; 0.77) p=0.87 | 0.04 (-0.91; 0.99) p=0.93 |
| Adults^b^ | 1.59 (0.05; 52.84) p=0.80 | 0.37 (0.02; 5.42) p=0.47 | 0.23 (0.01; 8.30) p=0.42 |  |  |  |
| Older adults^b^ | 1.73 (0.29; 10.14) p=0.55 | 1.06 (0.19; 5.88) p=0.95 | 0.61 (0.10; 3.78) p=0.60 |  |  |  |

Notes. Time by park interaction with ‘park = control’ and underlined time moment as reference categories. OR = odds ratio, CI = confidence interval.
^a^Gamma model with identity link, ^b^Hurdle model with part one = logistic regression and part two = gamma model with identity link. Bold results represent intervention effects.

# Intervention effects for the number of park visitors observed walking

The estimates of the total number of park visitors observed walking can be interpreted as the average difference between the intervention and control park in the change in number of visitors observed walking from pre-test to all post-intervention timepoints. The total number of visitors observed walking increased from pre-test to post-test with on average 0.87 (95% CI: 0.05; 1.69) visitors/ha more in the intervention park, compared to the control park. Between pre-test and follow-up one, this was on average 0.89 (95% CI: -0.01; 1.78) visitors/ha, and between pre-test and follow-up two this was 0.35 (95% CI: -0.43; 1.14) visitors/ha. No evidence of a change was found between anu of the post-intervention timepoints for the total number of park visitors observed walking.

Table 2: Intervention effects for the number of park visitors observed walking.

|  | Intervention effect for the odds of observing any visitor of the specified category walking | | | Intervention effects when at least one visitor of the specified category is observed walking | | | |
| --- | --- | --- | --- | --- | --- | --- | --- |
|  | **Pre-test – Post-test**  Time x Park OR (95% CI)  p value | **Pre-test – Follow-up 1**  Time x Park OR (95% CI)  p value | **Pre-test – Follow-up 2**  Time x Park OR (95% CI)  p value | | **Pre-test – Post-test**  Time x Park beta (95% CI)  p value | **Pre-test – Follow-up 1**  Time x Park beta (95% CI)  p value | **Pre-test – Follow-up 2**  Time x Park beta (95% CI)  p value |
| Children^b^ | 2.37 (0.43; 13.01) p=0.32 | 3.09 (0.52; 18.36) p=0.22 | 1.47 (0.24; 8.95)  p=0.68 | | -0.07 (-1.39; 1.26) p=0.92 | 0.11 (-1.25; 1.48)  p=0.87 | -0.05 (-1.40; 1.29)  p=0.94 |
|  | **Post-test – Follow-up 1**  Time x Park OR (95% CI)  p value | **Post-test – Follow-up 2**  Time x Park OR (95% CI)  p value | **Follow-up 1 – Follow-up 2**  Time x Park OR (95% CI)  p value | | **Post-test – Follow-up 1**  Time x Park beta (95% CI)  p value | **Post-test – Follow-up 2**  Time x Park beta (95% CI)  p value | **Follow-up 1 – Follow-up 2**  Time x Park beta (95% CI)  p value |
| Children^b^ | 1.30 (0.27; 6.37) p=0.75 | 0.62 (0.12; 3.12) p=0.56 | 0.48 (0.09; 2.61) p=0.39 | | 0.78 (-0.56; 0.92) p=0.63 | 0.01 (-0.68; 0.71) p=0.98 | -0.17 (-0.93; 0.60) p=0.66 |

Notes. Time by park interaction with ‘park = control’ and underlined time moment as reference categories. OR = odds ratio, CI = confidence interval.
^a^Gamma model with identity link, ^b^Hurdle model with part one = logistic regression and part two = gamma model with identity link. Bold results represent intervention effects.

# Intervention effects for the number of park visitors engaged in vigorous PA

Table 3: Intervention effects for the number of visitors observed engaged in vigorous activity.

|  | Intervention effect for the odds of observing any visitor of the specified category engaged in vigorous PA | | | Intervention effects when at least one visitor of the specified category is observed engaged in vigorous PA | | | |
| --- | --- | --- | --- | --- | --- | --- | --- |
|  | **Pre-test – Post-test**  Time x Park OR (95% CI)  p value | **Pre-test – Follow-up 1**  Time x Park OR (95% CI)  p value | **Pre-test – Follow-up 2**  Time x Park OR (95% CI)  p value | | **Pre-test – Post-test**  Time x Park beta (95% CI)  p value | **Pre-test – Follow-up 1**  Time x Park beta (95% CI)  p value | **Pre-test – Follow-up 2**  Time x Park beta (95% CI)  p value |
| Adults^a^ |  |  |  | | 0.40 (-0.05; 0.84) p=0.08 | 0.12 (-0.31; 0.55) p=0.59 | 0.30 (-0.15; 0.75) p=19 |
|  | **Post-test – Follow-up 1**  Time x Park OR (95% CI)  p value | **Post-test – Follow-up 2**  Time x Park OR (95% CI)  p value | **Follow-up 1 – Follow-up 2**  Time x Park OR (95% CI)  p value | | **Post-test – Follow-up 1**  Time x Park beta (95% CI)  p value | **Post-test – Follow-up 2**  Time x Park beta (95% CI)  p value | **Follow-up 1 – Follow-up 2**  Time x Park beta (95% CI)  p value |
| Adults^a^ |  |  |  | | -0.28 (-0.73; 0.18) p=0.23 | -0.10 (-0.57; 0.38) p=0.69 | 0.18 (-0.28; 0.64) p=0.44 |

Notes. Time by park interaction with ‘park = control’ and underlined time moment as reference categories. PA = physical activity, OR = odds ratio, CI = confidence interval.
^a^Gamma model with identity link, ^b^Hurdle model with part one = logistic regression and part two = gamma model with identity link. Bold results represent intervention effects.

# Intervention effects for the mean PA activity levels of the park visitors

Table 4: Intervention effects for the MET scores of the park visitors.

|  | Time x Park  **Pretest – Post-test** beta (95% CI)  p value | Time x Park  **Pretest – Follow-up 1** beta (95% CI)  p value | Time x Park  **Pretest – Follow-up 2**  beta (95% CI)  p value | Time x Park  **Post-test – Follow-up 1**  beta (95% CI)  p value | Time x Park  **Post-test – Follow-up 2**  beta (95% CI)  p value | Time x Park  **Follow-up 1 – Follow-up 2**  beta (95% CI)  p value |
| --- | --- | --- | --- | --- | --- | --- |
| Total^c^ | -0.25 (-0.97; 0.46)  p=0.48 | **-0.86 (-1.59; -0.14)**  **p=0.02** | -0.54 (-1.27; 0.19)  p=0.14 | -0.61 (-1.32; 0.10) p=0.09 | -0.29 (-1.00; 0.42) p=0.43 | 0.32 (-0.40; 1.04) p=0.38 |
| Children^c^ | 1.13 (-0.34; 2.59)  p=0.13 | 0.70 (-0.87; 2.26)  p=0.38 | 1.28 (-0.22; 2.78)  p=0.09 | -0.43 (-1.83; 0.97) p=0.54 | 0.15 (-1.17; 1.48) p=0.82 | 0.58 (-0.85; 2.02) p=0.42 |
| Adolescents^c^ | 0.31 (-1.09; 1.71)  p=0.66 | -1.09 (-2.55; 0.37)  p=0.14 | -1.13 (-2.69; 0.42)  p=0.15 | -1.40 (-2.83; 0.02) p=0.05 | -1.45 (-2.97; 0.07) p=0.06 | -0.04 (-1.62; 1.53) p=0.96 |
| Adults^c^ | -0.44 (-1.31; 0.43)  p=0.32 | -0.76 (-1.65; 0.12)  p=0.09 | -0.52 (-1.41; 0.36)  p=0.25 | -0.32 (-1.18; 0.54) p=0.46 | -0.08 (-0.94; 0.78) p=0.85 | 0.24 (-063; 1.11)  p=0.59 |
| Older adults^c^ | -0.31 (-1.68; 1.06)  p=0.66 | **-1.65 (-3.00; -0.29)**  **p=0.02** | -0.37 (-1.69; 0.96)  p=0.59 | **-1.34 (-2.67; -0.01) p=0.05** | -0.06 (-1.36; 1.24)  p=93 | 1.28 (-0.01; 2.57)  p=0.05 |

Notes. Time by park interaction with ‘park = control’ and underlined time moment as reference categories.
^c^Gaussian model with identity link.
Bold results represent intervention effects.
