# Supplementary material for: Evaluating the impact of park renovation on park-based physical activity: a natural experiment in Belgium with two years of follow-up
Source: Int J Behav Nutr Phys Act. 2025 Dec 5;22:154. doi: 10.1186/s12966-025-01846-0 (PMC12681091; doi:10.1186/s12966-025-01846-0)
Supplement: Supplementary file 4 — Supplementary Material 4. [file 12966_2025_1846_MOESM4_ESM.docx]

Supplementary File 4

Table 1: Descriptive statistics of the observed park visitors per activity level at pretest, post-test, follow-up 1 and follow-up 2.

|  | Intervention park | | | | Control park | | | | |  |  |
| --- | --- | --- | --- | --- | --- | --- | --- | --- | --- | --- | --- |
|  | Pretest | Post-test | Follow-up 1 | Follow-up 2 | Pretest | Post-test | Follow-up 1 | Follow-up 2 | |  |  |
| **Total number of park visitors** | 425 | 1316 | 1492 | 1248 | 412 | 347 | 333 | 300 | |  |  |
| **Park visitors observed sedentary (%)** | | | | | | | | | |  |  |
| Total | 123 | 416 | 636 | 570 | 104 | 92 | 56 | | 66 | | |
| Child | 16 (13%) | 126 (30%) | 100 (16%) | 75 (13%) | 19 (18%) | 37 (40%) | 6 (11%) | | 19 (29%) | | |
| Adolescents | 30 (24%) | 51 (12%) | 51 (8%) | 26 (5%) | 41 (39%) | 19 (21%) | 11 (20%) | | 4 (6%) | | |
| Adults | 67 (54%) | 209 (50%) | 351 (55%) | 322 (56%) | 34 (33%) | 30 (33%) | 34 (61%) | | 37 (56%) | | |
| Older adults | 10 (8%) | 30 (7%) | 134 (21%) | 147 (26%) | 10 (10%) | 6 (7%) | 5 (9%) | | 6 (9%) | | |
| **Park visitors observed walking (%)** | | | | | | | | | |  |  |
| Total | 119 | 355 | 411 | 252 | 88 | 70 | 99 | | 95 | |  |
| Child | 34 (29%) | 61 (17%) | 59 (14%) | 20 (8%) | 21 (24%) | 19 (27%) | 11 (11%) | | 10 (11%) | |  |
| Adolescents | 23 (19%) | 29 (8%) | 51 (12%) | 11 (4%) | 26 (30%) | 11 (16%) | 34 (34%) | | 4 (4%) | |  |
| Adults | 53 (45%) | 213 (60%) | 239 (58%) | 179 (71%) | 22 (25%) | 33 (47%) | 44 (44%) | | 65 (68%) | |  |
| Older adults | 9 (8%) | 52 (15%) | 62 (15%) | 42 (17%) | 19 (22%) | 7 (10%) | 10 (10%) | | 16 (17%) | |  |
| **Park visitors observed engaged in vigorous physical activity (%)** | | | | | | | | | |  |  |
| Total | 183 | 545 | 445 | 426 | 220 | 185 | 178 | | 139 | |  |
| Child | 23 (13%) | 141 (26%) | 154 (35%) | 94 (22%) | 60 (27%) | 50 (27%) | 34 (19%) | | 21 (15%) | |  |
| Adolescents | 27 (15%) | 89 (16%) | 72 (16%) | 47 (11%) | 69 (31%) | 35 (19%) | 46 (26%) | | 22 (16%) | |  |
| Adults | 122 (67%) | 278 (51%) | 197 (44%) | 247 (58%) | 69 (31%) | 81 (44%) | 74 (42%) | | 76 (55%) | |  |
| Older adults | 11 (6%) | 37 (7%) | 22 (5%) | 38 (9%) | 22 (10%) | 19 (10%) | 24 (13%) | | 20 (14%) | |  |

Table 2: mean and SD of the number of visitors per hectare according to activity level and timepoint.

|  | Intervention Park | | | | Control Park | | | |
| --- | --- | --- | --- | --- | --- | --- | --- | --- |
|  | Pre-test  Mean (SD) | Post-test  Mean (SD) | Follow-up 1  Mean (SD) | Follow-up 2  Mean (SD) | Pre-test  Mean (SD) | Post-test  Mean (SD) | Follow-up 1  Mean (SD) | Follow-up 2 Mean (SD) |
| Total park visitors/hectare | 2.30 (2.08) | 5.46 (4.13) | 6.55 (4.33) | 5.48 (3.73) | 3.63 (3.60) | 2.78 (3.53) | 2.94 (3.82) | 2.89 (2.19) |
| **Park visitors observed sedentary (n/hectare)** | | | | | | | | |
| Total | 1.00 (0.66) | 1.97 (2.20) | 3.46 (2.08) | 3.00 (2.10) | 1.32 (1.20) | 1.08 (1.34) | 0.77 (0.77) | 1.00 (1.03) |
| Children | 0.13 (0.23) | 0.60 (0.67) | 0.54 (0.52) | 0.39 (0.44) | 0.24 (0.71) | 0.44 (1.00) | 0.08 (0.22) | 0.29 (0.57) |
| Adolescents | 0.24 (0.35) | 0.24 (0.47) | 0.28 (0.37) | 0.14 (0.28) | 0.52 (1.03) | 0.22 (0.29) | 0.15 (0.34) | 0.06 (0.19) |
| Adults | 0.54 (0.43) | 0.99 (1.25) | 1.91 (1.20) | 1.69 (1.08) | 0.43 (0.48) | 0.35 (0.41) | 0.47 (0.55) | 0.56 (0.55) |
| Older adults | 0.08 (0.21) | 0.14 (0.20) | 0.73 (1.03) | 0.77 (1.07) | 0.13 (0.26) | 0.07 (0.16) | 0.07 (0.16) | 0.09 (0.18) |
| **Park visitors observed walking (n/hectare)** | | | | | | | | |
| Total | 0.83 (1.25) | 1.51 (1.25) | 1.85 (1.20) | 1.11 (0.81) | 1.12 (1.16) | 0.93 (0.74) | 1.26 (1.76) | 1.04 (1.04) |
| Children | 0.24 (0.85) | 0.26 (0.33) | 0.27 (0.42) | 0.09 (0.17) | 0.27 (0.80) | 0.25 (0.49) | 0.14 (0.37) | 0.11 (0.32) |
| Adolescents | 0.16 (0.37) | 0.12 (0.24) | 0.23 (0.30) | 0.05 (0.13) | 0.33 (0.44) | 0.15 (0.23) | 0.43 (1.18) | 0.04 (0.24) |
| Adults | 0.37 (0.28) | 0.91 (0.70) | 1.08 (0.81) | 0.79 (0.48) | 0.28 (0.29) | 0.44 (0.44) | 0.56 (0.50) | 0.71 (0.75) |
| Older adults | 0.06 (0.11) | 0.22 (0.39) | 0.28 (0.26) | 0.18 (0.31) | 0.24 (0.36) | 0.09 (0.22) | 0.13 (0.29) | 0.18 (0.26) |
| **Park visitors observed engaged in vigorous PA (n/hectare)** | | | | | | | | |
| Total | 1.08 (0.73) | 2.38 (1.56) | 2.01 (1.34) | 1.92 (1.31) | 2.18 (2.12) | 1.78 (2.02) | 1.77 (2.02) | 1.42 (1.17) |
| Children | 0.14 (0.29) | 0.62 (1.02) | 0.69 (0.85) | 0.42 (0.52) | 0.60 (1.09) | 0.48 (1.29) | 0.34 (1.13) | 0.22 (0.69) |
| Adolescents | 0.16 (0.24) | 0.39 (0.50) | 0.32 (0.43) | 0.21 (0.34) | 0.68 (1.40) | 0.34 (0.63) | 0.46 (1.06) | 0.23 (0.34) |
| Adults | 0.72 (0.49) | 1.21 (0.73) | 0.89 (0.40) | 1.11 (0.98) | 0.68 (0.68) | 0.78 (0.74) | 0.73 (0.58) | 0.78 (0.62) |
| Older adults | 0.06 (0.11) | 0.16 (0.18) | 0.10 (0.16) | 0.17 (0.16) | 0.22 (0.25) | 0.18 (0.26) | 0.24 (0.34) | 0.20 (0.29) |

Notes: SD = standard deviation, PA = physical activity.
